# Genome Analysis of Planctomycetes Inhabiting Blades of the Red Alga *Porphyra umbilicalis*

**DOI:** 10.1371/journal.pone.0151883

**Published:** 2016-03-25

**Authors:** Jay W. Kim, Susan H. Brawley, Simon Prochnik, Mansi Chovatia, Jane Grimwood, Jerry Jenkins, Kurt LaButti, Konstantinos Mavromatis, Matt Nolan, Matthew Zane, Jeremy Schmutz, John W. Stiller, Arthur R. Grossman

**Affiliations:** 1 Department of Biomolecular Engineering, University of California Santa Cruz, Santa Cruz, California, United States of America; 2 School of Marine Sciences, University of Maine, Orono, Maine, United States of America; 3 Department of Energy, Joint Genome Institute, Walnut Creek, California, United States of America; 4 HudsonAlpha Institute for Biotechnology, Huntsville, Alabama, United States of America; 5 Department of Biology, East Carolina University, Greenville, North Carolina, United States of America; 6 Department of Plant Biology, Carnegie Institution for Science, Stanford, California, United States of America; Illinois Institute of Technology, UNITED STATES

## Abstract

*Porphyra* is a macrophytic red alga of the Bangiales that is important ecologically and economically. We describe the genomes of three bacteria in the phylum Planctomycetes (designated P1, P2 and P3) that were isolated from blades of *Porphyra umbilicalis* (P.um.1). These three Operational Taxonomic Units (OTUs) belong to distinct genera; P2 belongs to the genus *Rhodopirellula*, while P1 and P3 represent undescribed genera within the Planctomycetes. Comparative analyses of the P1, P2 and P3 genomes show large expansions of distinct gene families, which can be widespread throughout the Planctomycetes (e.g., protein kinases, sensors/response regulators) and may relate to specific habitat (e.g., sulfatase gene expansions in marine Planctomycetes) or phylogenetic position. Notably, there are major differences among the Planctomycetes in the numbers and sub-functional diversity of enzymes (e.g., sulfatases, glycoside hydrolases, polysaccharide lyases) that allow these bacteria to access a range of sulfated polysaccharides in macroalgal cell walls. These differences suggest that the microbes have varied capacities for feeding on fixed carbon in the cell walls of P.um.1 and other macrophytic algae, although the activities among the various bacteria might be functionally complementary *in situ*. Additionally, phylogenetic analyses indicate augmentation of gene functions through expansions arising from gene duplications and horizontal gene transfers; examples include genes involved in cell wall degradation (e.g., κ-carrageenase, alginate lyase, fucosidase) and stress responses (e.g., efflux pump, amino acid transporter). Finally P1 and P2 contain various genes encoding selenoproteins, many of which are enzymes that ameliorate the impact of environmental stresses that occur in the intertidal habitat.

## Introduction

Marine macroalgae and bacteria have varied and complex interactions [1]. Remarkably, the red macroalga *Delisea pulchra* foils attack from a proteobacterium by producing furanones that inhibit quorum-sensing molecules (N-acyl homoserine lactones, AHLs) used for bacterial communication [2]. In contrast, swimming zoospores of the green alga *Ulva* select settlement sites by sensing AHLs produced by some bacteria [3]. For heterotrophic bacterial “farmers” [4], macroalgal cell walls are a carbon-rich habitat, while bacterial symbionts may synthesize plant growth regulators that stabilize macroalgal morphology [5–7] and provide the algae with inorganic nutrients and vitamins {e.g., [8]}. Presently, only one symbiotic association has been characterized in some detail {i.e. [9]}.

Little is known as to why different bacteria colonize different algae, and the nature of the complex and dynamic interactions between them [10]. Sympatric macroalgae growing together can harbor substantially different proportions of bacterial phyla. Phyletic effects on the bacterial composition can be larger than observed seasonal or biogeographic impacts [11–13], suggesting that bacteria have selective abilities to feed on different algal cell wall types. Cell wall composition varies among the marine Chlorophyta [cellulose, xyloglucan, mannan, glucuronan, (1,3) β-glucan, ulvan], Rhodophyta [cellulose, (1,4) β-D-mannan, (1,4) β-D-xylan, (1,3) β-D-xylan, glucomannan, sulfated MLG, (1,3) (1,4) β-D-xylan, agars, porphyran, carrageenans] and Phaeophyceae [cellulose, sulfated xylofucoglucuronan, (1,3) β-glucan, alginates (polymannuronic acid, polyguluronic acid), homofucans] [14]. Moreover, within the Rhodophyta, the cell walls in different phases of the life histories (e.g., gametophyte/sporophyte) can show variations in their compositions [14–16].

Examination of the coding capacity of different bacteria for enzymes that degrade cell wall moieties informs our understanding of microbial/macroalgal ecology and evolution. Red algal cell walls are composed mostly of the sulfated polymers porphyran, agar and/or carrageenan, in addition to some xylan and/or cellulose microfibrils [14]. The biosynthesis and degradation of sulfated cell wall polysaccharides of macrophytic algae requires several enzymes including glycoside hydrolases (GHs), sulfatases and carbohydrate sulfotransferases. A range of such enzymes are encoded on many marine bacterial genomes. Indeed, the marine bacterium *Zobellia galactanivorans* (Bacteroidetes) has a genome encoding 130 GHs, 12 polysaccharide lyases and 71 sulfatases (Genoscope: **G0L495**), and is being developed as a model for producing enzymes that function in bioconversion of algal polysaccharides. This bacterium is associated with green algae [17,18], red algae [19], brown algae [17] and dinoflagellates [20], and has been examined in detail for its ability to synthesize enzymes capable of degrading sulfated galactans of the red macrophyte *Delesseria sanguinea*, with specific characterizations of *β*-agarases [21–23], *κ*- and *ι*-carrageenases [24,25] and porphyranases [26,27].

Recently, attention has focused on another macroalgal-associated bacterial phylum, the Planctomycetes. These bacteria are usually a smaller proportion of the macroalgal associated bacteria than Bacteroidetes or Proteobacteria, but may account for 50% of the bacteria on some brown algae {e.g., [28]}. The Planctomycetes are part of the Planctomycetes-Verrucomicrobia-Chlamydiae (PVC) superphylum [29,30], including some genera that can synthesize a large number of hydrolytic enzymes [31,32]. They exhibit unusual features for bacteria, including division by budding, endocytosis with coated vesicles, a wall composed primarily of glutamine-rich glycoproteins and extensive invaginations of the inner membrane [33–35]. Further, many planctomycete genes are not organized into operons [31], and some encode proteins more typically found in eukaryotes [36].

In a recent study [4], bacterial diversity on the blades of *Porphyra umbilicalis* (Rhodophyta) was analyzed from wild plants and antibiotic-treated, laboratory-cultures. Eight phyla were identified (Bacteroidetes, Proteobacteria, Planctomycetes, Chloroflexi, Actinobacteria, Deinococcus-thermus, Firmicutes, and the candidate division TM7), with the majority of sequences from both field and laboratory material coming from the Bacteroidetes. The abundance of blade-associated Planctomycetes was small on wild blades (0.03–1.1%), but enriched (4.06%,) when *P*. *umbilicalis* {strain P.um.1 [37]} was treated with antibiotics that eliminate most bacteria. Four planctomycete OTUs were enriched: *Rhodopirellula baltica* and three undescribed planctomycetes. We have assembled the genomes of these three undescribed planctomycetes and examine their phylogenetic affiliations, genome structures and functional potential.

## Materials and Methods

### Sample collection

The P.um.1 isolate was collected at Schoodic Point, Maine (44°20’1.68” N; 68°3’29.14”W) on April 3, 2008 [4,37,38]. Details regarding sample preparation are available in S1 Text. Scientific research and collecting permits authorizing field studies pertaining to the P.um.1 isolate were obtained from the United States Department of the Interior, National Park Service, Acadia National Park (permit #s: ACAD-2008, 2009, 2010, 2011-SCI-0004). These field studies did not involve protected or endangered species.

### Genome sequencing and assembly

The 454 sequencing was performed on standard (500–800 bp) and long distance (10 kb) paired-end, genomic libraries (S1 Text). The three largest scaffolds (8.5, 7.3 and 3.8 Mbp) from a preliminary assembly with Newbler (v.2.3-PreRelease-10/20/2009, Roche) were microbial based on sequence similarities in the NCBI (nr) database. We performed additional Illumina sequencing to correct 454 homopolymer errors in the three scaffolds and reassembled the 3.8 Mbp scaffold into a 4.9 Mbp scaffold because it appeared to be an incomplete genome based on its gene complement (S1 Text). These three large scaffolds correspond to genomes of Planctomycetes that we designated P1 (8.5 Mbp), P2 (7.3 Mbp) and P3 (4.9 Mbp).

### Genome annotation

The three scaffolds were first annotated through the Joint Genome Institute’s microbial annotation pipeline and deposited in the Integrated Microbial Genomes (IMG) database (http://img.jgi.doe.gov/). Additional annotations were conducted for genes of interest with missing functional annotations, protein-coding gene families, repetitive DNA elements, transposable element (TE)-associated genes, selenoproteins and selenocysteine utilization elements, and genomic islands. See S1 Text for additional information.

### Phylogenetic analyses

An initial phylogeny based on 16S rDNA sequences for 25 bacterial species was generated using RAxML [39] with the GTR-GAMMA model. A more robust phylogeny was built by sampling across multiple protein-coding loci [40] corresponding to 39 single-copy genes encoding highly conserved proteins (S1 Table and S1 Text). Homologs for the 39 genes from each of the 23 genomes studied (S2 Table) were aligned, trimmed and then concatenated adhering to a predetermined, randomized gene order. A maximum-likelihood (ML) phylogeny based on 8,725 amino acid positions was inferred from 1000 bootstrap iterations using RAxML. All protein-coding gene trees (see Results) were generated using a similar procedure (S1 Text).

### Classification of sulfatases and carbohydrate active enzymes

Sulfatase subclasses were determined based on clades in ML phylogenies of all sulfatase sequences for a given organism. Each resolvable clade was annotated as iduronate-2-sulfatase, heparan-N-sulfatase, mucin-desulfating sulfatase or choline sulfatase, based on BLASTp hits against UNIPROT TREMBL [41]. Unresolvable sulfatases were placed in the more general categories ‘arylsulfatase A’ and ‘galactosamine-N-acetyl-6-sulfatases’ (GALNS). We identified hydrolytic enzymes in the Carbohydrate-Active enZYmes (CAZY) database (http://www.cazy.org) using the CAZY Analysis Toolkit, which executes a BLASTp search against the CAZY database. Hits to the genomes used for our analysis had e-values of <10^−10^.

### Identification of genes encoding selenoproteins and Sec insertion and utilization elements

The Sec-insertion and utilization genes (*selA*, *selB*, *selD*, *ybbB*) were identified by sequence alignments (BLASTp) against known bacterial homologs. Genes potentially encoding selenoproteins were identified on the basis of in-frame opal (‘UGA’) stop codons, homology searches against known selenoproteins and the presence of SECIS elements. See S1 Text for additional information.

## Results and Discussion

### Genome assembly validation and phylogeny

The bacterial strains used here, including the three novel planctomycete genomes recovered from the P.um.1 sequenced libraries are given in S2 Table. Properties of P1 (8.5 Mb), P2 (7.3 Mb) and P3 (4.9 Mb) are provided in Table 1 along with tRNA gene predictions for 29 bacterial genomes, including 22 species from the PVC superphylum, in S3 Table.

**Table 1:** Properties of 9 bacterial genome assemblies including 8 Planctomycetes and 1 marine Bacteroidetes. P1, P2 and P3 were sequenced from a blade of *Porphyra umbilicalis*. Strains of *R*. *baltica*, *P*. *mikurensis* and *Z*. *galactanivorans* (Bacteroidetes) were also present on the blade (based on 16S rDNA analysis). *P*. *staleyi*, *R*. *maiorica* and *B*. *marina* are the closest known relatives of P1, P2 and P3, respectively.

	*R*. *baltica*	*R*. *maiorica*	P2	P1	*P*. *staleyi*	*B*. *marina*	P3	*P*. *mikurensis*	*Z*. *galactanivorans*
Genome assembly size (kb)	7146	8874	7267	8470	6196	6654	4918	3803	5522
Number of scaffolds	1	1132	1	1	1	64	1	1	1
Estimated size of gaps (kb)	0	0	242	205	0	0	15	0	0
GC content (%)	55.4	54.7	54.9	49.1	57.5	57	61.7	73.3	42.8
Protein-coding genes	7325	7825	5409	6382	4773	6025	4088	3201	4732
rRNA genes	3	3	3	4	3	9	4	3	6
tRNA genes	76	80	51	54	46	53	96	46	40
Other RNA genes	10	**	11	3	3	6	3	2	9
Tandem repeat content (repeat bases / kb of genome)	12.2	18.7	12.7	10.8	16.4	10	19.7	93.5	19.3
TE-associated genes	85	48	26	32	28	75	84	16	28
CRISPRs									
Confirmed	0	0	1	0	2	1	1	2	1
Questionable*	2	11	6	6	0	4	4	13	1

* Small CRISPRs with only two or three direct repeats or CRISPR structures where direct repeats are not 100% identical.

** Data not available in the Integrated Microbial Genomes database.

**Fig 1:**
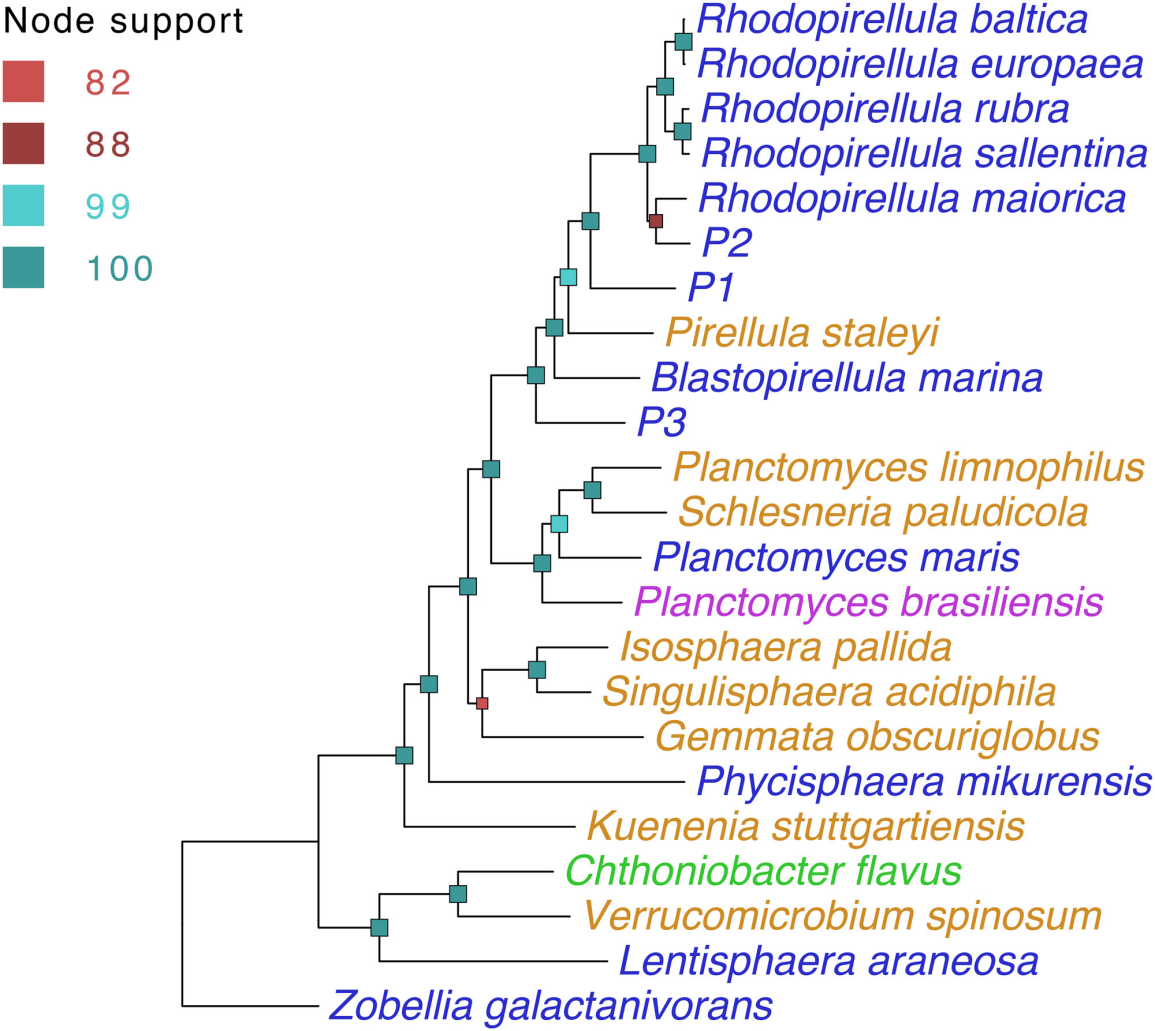
Phylogeny of three novel planctomycetes and related species. The phylogeny shown is based on concatenated protein-coding sequences of 39 highly conserved, single-copy genes (see S1 Table). Consensus maximum likelihood trees from 1000 bootstrap iterations are shown. Internal nodes are color-coded (indicated to the left of each tree) based on bootstrap support values. Taxa are color-coded by the type of habitat from which they were isolated: marine [blue], freshwater [orange], marine/brackish [purple], soil [green].

For phylogenetic classification, we constructed a high resolution [40] ML tree (Fig 1) based on 39 ‘core’ protein-coding genes (S1 Table). The three sequenced genomes are part of a clade that includes the genera *Blastopirellula*, *Pirellula* and *Rhodopirellula*. P3 is recovered as the most ancestral taxon in this clade, while P2 appears to be an undescribed OTU within the genus *Rhodopirellula*, and P1 shares a direct common ancestor with the *Rhodopirellula* sub-clade. A ML tree based on 16S rDNA (S1 Fig) indicates consistent phylogenetic positions for P1, P2 and P3. P1 and P3 represent new Planctomycetes’ genera based on 16S rDNA sequence analysis (S4 Table).

### Gene functions and gene family content

The P1, P2 and P3 genomes are non-syntenic with those of other sequenced planctomycete genomes (S2 Fig), and previous work showed that gene content is better preserved than synteny among the Planctomycetes [34]. Many planctomycete genomes have extensive expansions of protein-coding gene families {e.g., sulfatases in *Rhodopirellula* [32]}; this is also the case for P1, P2 and P3 (S5 Table). Within the Planctomycetes, the percentage of genes belonging to gene families (2 or more) ranged from 36% in *P*. *mikurensis* to 59% in *S*. *acidiphila*. Previous studies reported a linear relationship between genome size and percentage of genes in families [42,43]. While most genomes that we analyzed followed this trend, there were several outliers (S3 Fig). Some of the *Rhodopirellula* and P1 have low densities of gene families despite their large genomes, while *K*. *stuttgartiensis* has high gene family density for a small genome (S2 Text).

Highly represented gene families are summarized in S6 Table, with the full list of families in S1 Data. The largest gene families encode response regulators (RR), serine/threonine protein kinases (STPK), transporters (ABC), sigma factors, sulfatases and solute-binding proteins with the 1559 domain of unknown function (DUF1559), which appears exclusive to the PVC superphylum. While some gene families are expanded throughout the Planctomycetes, others such as the sulfatases are more specific to phylogenetic position and/or the type of habitat in which the organism is found (e.g., relative number of sulfatase genes in marine vs. freshwater vs. anammox Planctomycetes).

**Fig 2:**
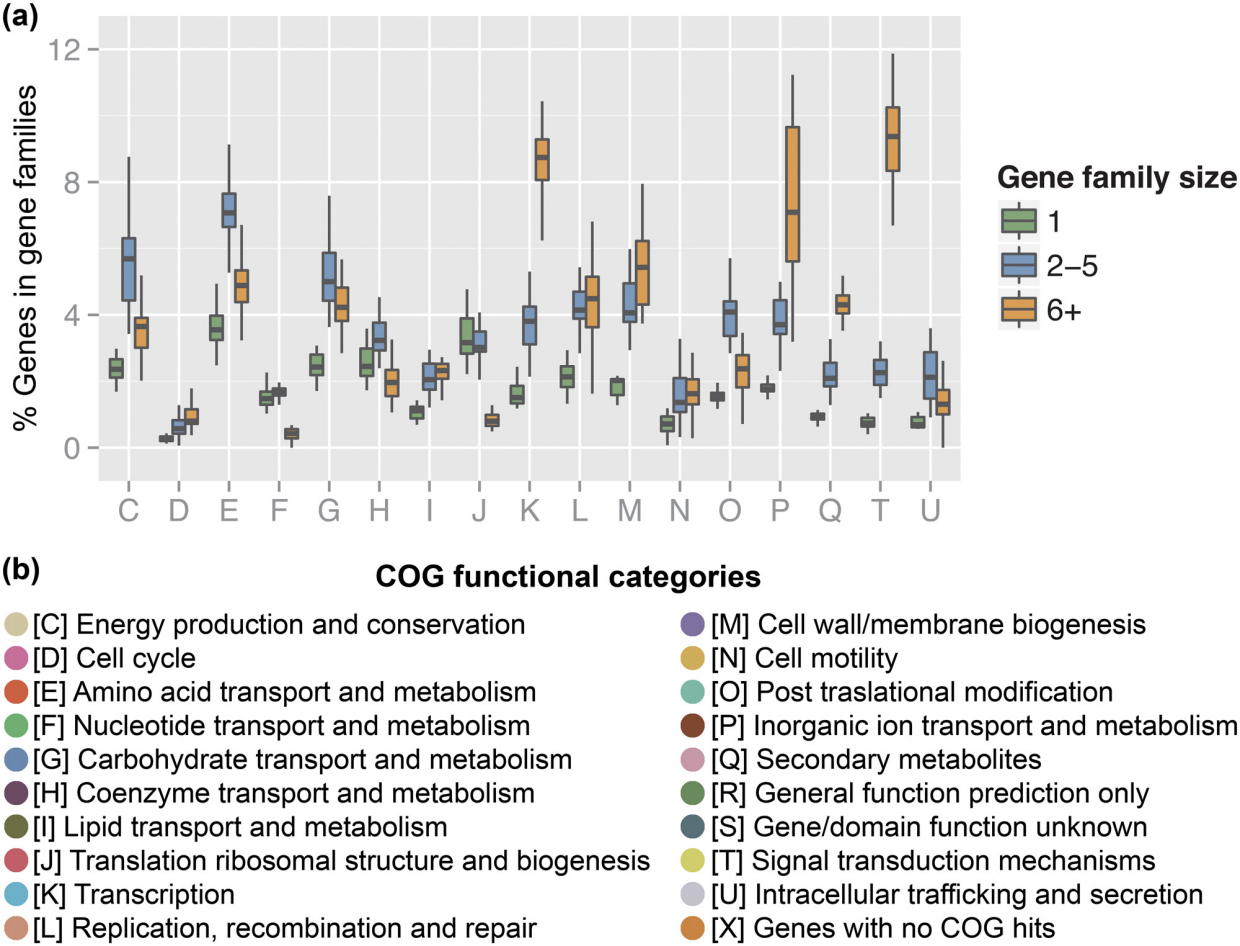
Distribution of COG functional categories in paralogous gene families. **(a)** Distribution across families containing only singletons, or with 2–5 members or 6+ members. Paralogous gene families were identified using a network-based approach (see S1 Text). **(b)** Definition of COG categories on the x-axis of **(a)** (and also in S4 Fig).

An investigation of the relationship between higher-level functional classification and gene family size across the 23 genomes studied shows relatively small variations in the COG functional distribution of singleton genes when compared to gene families with more than one member (Fig 2). The largest variation across 23 genomes is in the category ‘inorganic ion transport and metabolism’ (P), which contains the sulfatases. The absolute distribution of COG domain hits for P1, P2 and P3 is shown in S4 Fig. More in-depth data on gene families and higher-level functional classifications are in the S2 Text.

### The sulfatases

**Fig 3:**
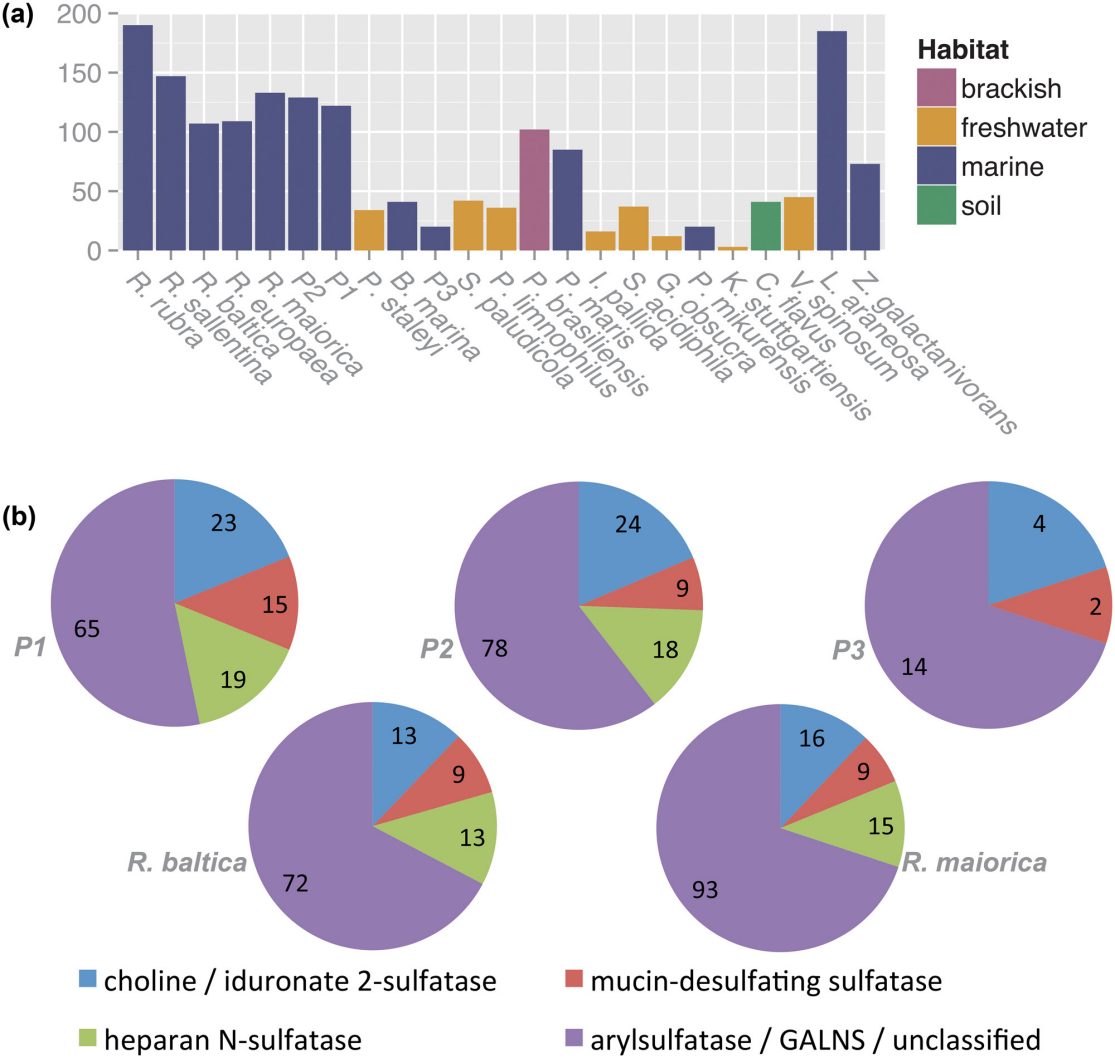
Sulfatase gene distribution and sub-classification in Planctomycetes and related strains. **(a)** Number of sulfatase genes in various Planctomycetes and related strains. Only sulfatase genes encoding the active site and ≥350 amino acid residues were included. **(b)** Functional subclasses of sulfatases present in P1, P2, P3, *R*. *baltica* and *R*. *maiorica*. For each organism, the total number of sulfatases (≥350 residues) is divided into the following subclasses: choline/iduronate-2-sulfatases, mucin-desulfating sulfatases, heparan-N-sulfatases, and unclassified sulfatases including general arylsulfatases and galactosamine N-acetyl-6-sulfate sulfatases.

Sulfatase genes comprise one of the largest families in the Planctomycetes, especially in the genus *Rhodopirellula* (Fig 3, S6 Table). Both sulfatases and GHs are needed for degrading algal cell walls, allowing bacteria to access fixed carbon in sulfated polysaccharides, which can make up in excess of 50% of the dry biomass of macrophytic algae [14,44,45]. Sulfatases catalyze the hydrolysis of sulfate esters and couple with sulfotransferases to facilitate both degradation and synthesis of compounds containing esterified sulfate. The various sulfatases, including alkyl- and arylsulfatases, can have distinct specificities, metabolizing sulfated carbohydrates, proteins and lipids, as well as sulfated glycosaminoglycans and glycolipids [46–48]. A diversity of carbohydrate sulfates can serve as sulfatase substrates, including polysaccharides in cell walls of marine macrophytic algae [27,49,50].

Various sulfatase types are encoded on the planctomycete genomes. Counting only “full-length” ORFs (encoding ≥350 amino acids and containing the active site), there are 122 putative sulfatases in P1, 129 in P2 and only 20 in P3; results for all 23 organisms in our analyses are given in Fig 3a. The active sites of sulfatases are defined by the sequence C/S-X-P-S/X-R-X-X-X-L/X-T/X-G/X-R/X, in which the cysteine is modified to a formylglycine. The various sulfatases are classified as iduronate-2-sulfatases, heparan-N-sulfatases, mucin-desulfating sulfatases, GALNS sulfatases, with many in the more general arylsulfatase category. The number of full-length sulfatases in each category, determined by phylogenetic analyses, is given in Fig 3b. Based on signal sequence predictions, 79, 91 and 10 sulfatases from P1, P2 and P3, respectively, enter the secretory pathway, likely accessing their substrates from the extracellular space. Enzymes involved in conversion of the sulfatase active site cysteine to a formyl-glycine [51] are also encoded on the P1, P2 and P3 genomes, with 7, 7 and 8 genes, respectively (S2 Text).

**Fig 4:**
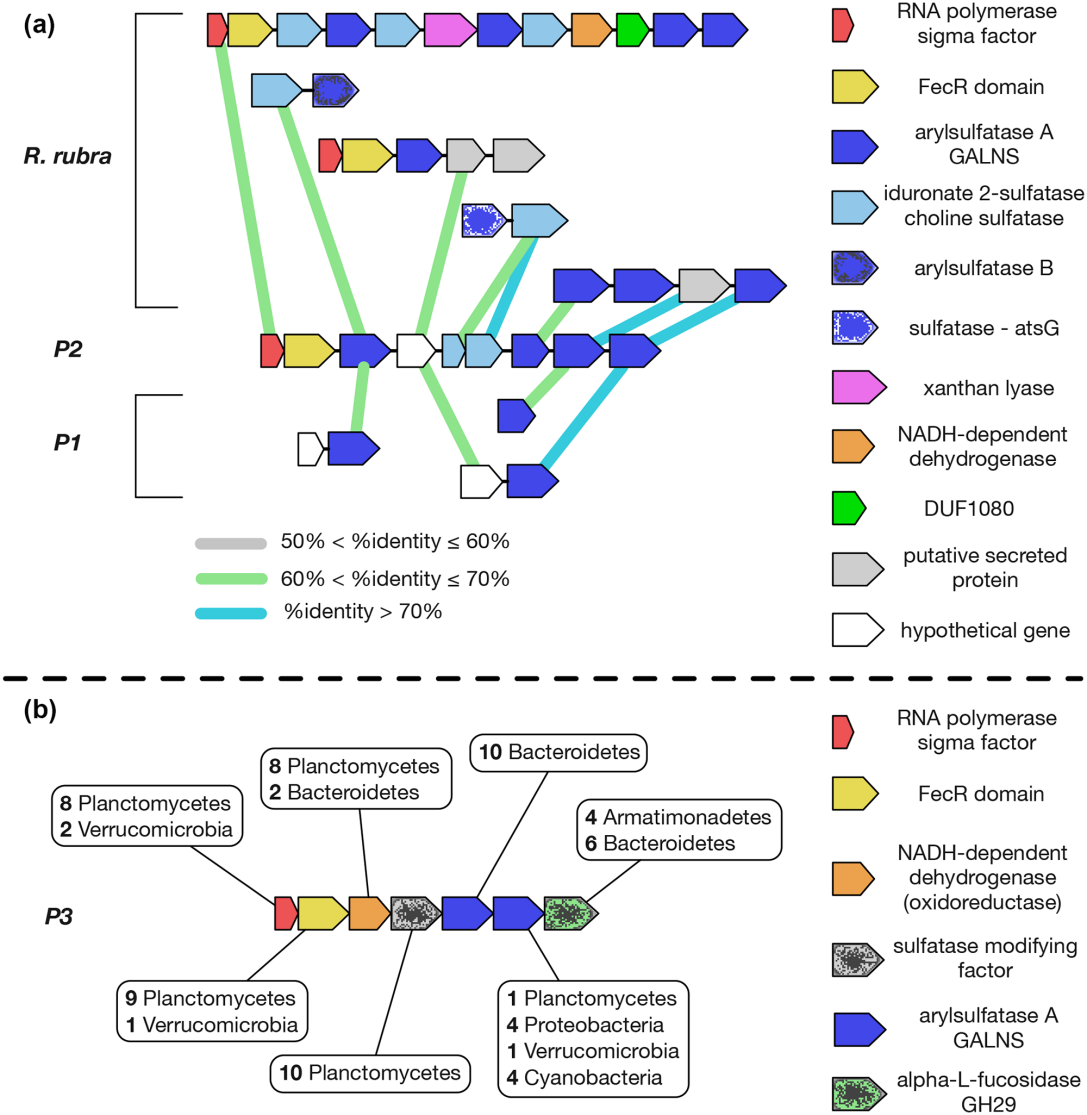
Changing context of sulfatase genes in operons. **(a)** Changing genetic context of individual sulfatase genes of a co-oriented P2 sulfatase gene cluster, resembling an operon. Adjacent genes are joined by a black line, and all genes are color-coded by predicted function as given on the right-hand side of the figure. P1 and *R*. *rubra* homologs for individual sulfatase genes in the P2 operon are shown. For each homolog, the immediate context of adjacent, co-oriented genes within their respective genomes is also shown. Reciprocal best-hit genes across organisms are connected by thick colored lines (gray, green, cyan). ORF lengths and intergenic distances are not drawn to scale. **(b)** A heterophyletic gene cluster resembling an operon in P3. Seven consecutive genes are color-coded by predicted function as given on the right-hand side of the figure. The distribution of top 10 BLASTp hits across various bacterial phyla is provided for each gene.

While the distribution of sulfatase genes on the P1, P2 and P3 genomes appears to be largely random, some occur in clusters resembling operons (Fig 4). In P1, P2 and P3 there are 10, 20 and 3 instances, respectively, where sulfatase genes reside at adjacent positions on the genome, with a single pair in P1 (IMG: 2643311965, 2643311966) that shows relatively high amino acid sequence identity (76%) and thus likely arose via a recent tandem duplication. The remaining adjacent pairs are dissimilar (avg. BLASTp sequence identity for P1, 29.0±6.1; P2, 27.6±3.7; P3, 26.7±3.4) and have significantly higher sequence identity to putative PVC orthologs than to each other (avg. BLASTp identity for P1, 64.0±12.7; P2, 68.1±10.6; P3, 51.2±9.9). Also, potential orthologs encoding adjacent P1, P2 and P3 sulfatases are rarely adjacent on the genomes of other closely related Planctomycetes. This suggests that most tandem arrangements of sulfatase genes in P1, P2 and P3 are the consequence of genomic rearrangements and/or HGT, rather than recent tandem duplications. Interestingly, the likelihood of finding even a single pair of adjacent sulfatase genes on the P1, P2 and P3 genomes is very small (permutation test with 10,000 permutations, P1, *p* = 0.0; P2, *p* = 0.0; P3, *p* = 0.0) assuming random genome rearrangements with no tandem duplications. This suggests that functional associations (e.g., co-expression of adjacent genes working together to degrade specific polysaccharides) could drive sulfatase gene clustering.

Interestingly, a gene containing two sulfatase domains is present in both P1 (IMG: 2643314295) and P2 (IMG: 2643291516), likely resulting from the fusion of two unrelated, adjacent sulfatase genes. The two ancestral domains of this gene appear to have different evolutionary origins; the protein encoded by the 5’ domain most closely resembles (~60% amino acid identity to P1 and P2 orthologs) an arylsulfatase A from the Verrucomicrobia bacterium SCGC AAA164-E04 (GI: 518992481), while the protein encoded by the 3’ domain is most similar (~50% amino acid identity to P1 and P2 orthologs) to an iduronate-2-sulfatase/choline sulfatase of *Saccharicrinis fermentans* (GI: 763406655) in the Bacteroidetes. We estimate based on protein length (>600 amino acids) that there are 34 and 21 sulfatase genes on P1 and P2 that encode multi-domain proteins, most often containing glycoside hydrolase and hypothetical protein domains, but also including alginate lyase, esterase/lipase, laminin G, and HEAT_2 repeat domains. Gene fusion appears to contribute to the evolution of multi-domain sulfatase genes, potentially pairing sulfatases with various other functions.

The expansion of the sulfatase gene family appears to be accompanied by high rates of genomic rearrangement {consistent with prior observations [34]} that can lead to innovation of protein function (e.g., domain swapping and gene fusion) as well as the generation and modification of operons (Fig 4a). In P1, P2 and P3, co-oriented gene clusters resembling operons are often heterophyletic (i.e. member genes with different evolutionary backgrounds). One such P3 gene cluster is shown in Fig 4b, in which member genes, including two sulfatase genes and an α-L-fucosidase gene, have highly discordant BLASTp hit distributions (across NCBI nr); the closest hits for individual members occur in the Bacteroidetes, Proteobacteria, Armatimonadetes and the Planctomycetes. Furthermore, there appears to be a high turnover rate of member genes within such clusters as evidenced by rearrangements of sulfatase genes between various planctomycete OTUs, even within the same genera (Fig 4a). Despite this high turnover rate, likely caused by random genomic rearrangements and HGT, genes encoding polysaccharide degradation enzymes are often found in clusters (e.g., adjacent sulfatase genes, Fig 4a and 4b). One possible explanation is that diversification of operons can confer an adaptive advantage, and is therefore selected.

### Polysaccharide degrading enzymes

Sulfated polysaccharides like agars, carrageenans and porphyrans have high proportions of galactose monomers within a polymeric hexose structure. The porphyran polymer, like agarose, has a backbone of repeating disaccharide units, but the disaccharide is a 3-linked β-D-galactosyl unit alternating with a 4-linked 3,6-anhydro-α-L-galactose. Some of the monomeric units are sulfated at the C6 position while others may be methylated [52]; this is not characteristic of agarose.

Based on P1, P2 and P3 genome sequences, these organisms can synthesize a large number of GHs and polysaccharide lyases (PLs) that have the potential to degrade both 1,3 and 1,4 hexose polymers. GH and PL subclasses that are abundant or over-represented in at least one of the three planctomycete isolates are given in Table 2, with descriptions of the subclasses in S7 Table. Many subclasses are also represented in other Planctomycetes, in members of *the larger* PVC superphylum, and in *Z*. *galactanivorans*. The distributions of genes across all CAZY families and subclasses for the 23 genomes are provided in S2 Data.

**Table 2:** Cell wall degradation enzymes in planctomycetes and related species. The number of BLASTp hits (e-value < 1e-10) is shown for selected GH and PL domains, which are involved in the degradation of algal, fungal, and vascular plant cell walls. The rows are ordered according to the phylogeny in Fig 1. Entries for P1, P2 and P3 are bolded in cases where the number of members within a CAZY category has a percent rank among all shown species that is greater than 75%.

	cellulases/xylanases			agarases/carrageenases/ galactanases/porphyranases				fucosidases		arabinases/ neoagarobiases		alginate lyases/ pectate lyases		
Organism	GH3	GH10	GH74	GH16	GH50	GH53	GH86	GH29	GH95	GH43	GH117	PL6	PL9	PL14
*Rhodopirellula rubra*	7	29	1	17	3	21	1	28	20	**43**	**44**	5	2	0
*Rhodopirellula sallentina*	4	20	3	15	2	18	4	23	24	50	31	0	2	1
*Rhodopirellula baltica*	9	17	0	4	0	15	1	1	1	27	12	0	1	1
*Rhodopirellula europaea*	14	18	0	4	0	15	1	1	1	31	15	0	0	2
*Rhodopirellula maiorica*	13	19	1	11	1	18	1	11	13	44	29	0	0	0
P2	8	**32**	0	4	1	9	0	2	1	**37**	14	1	**3**	**2**
P1	**25**	**31**	**4**	**18**	**4**	13	**2**	2	1	30	14	**3**	**4**	**4**
*Pirellula staleyi*	8	8	0	4	0	7	0	0	2	9	3	0	1	0
*Blastopirellula marina*	12	7	0	3	0	9	0	0	2	12	7	0	1	0
P3	11	4	1	**11**	1	11	1	**8**	**5**	7	4	1	0	0
*Schlesneria paludicola*	7	5	0	4	0	11	0	0	2	10	4	0	0	0
*Planctomyces limnophilus*	6	5	0	2	0	6	0	0	0	7	6	0	1	0
*Planctomyces brasiliensis*	10	13	0	1	1	8	0	1	0	29	14	0	0	0
*Planctomyces maris*	8	9	0	1	1	8	0	0	2	20	7	1	3	0
*Isosphaera pallida*	6	1	1	4	0	5	0	1	2	4	2	0	2	0
*Singulisphaera acidiphila*	21	5	1	3	0	11	0	0	3	12	2	0	0	0
*Gemmata obscuriglobus*	11	3	0	6	0	0	0	0	0	5	2	0	1	0
*Phycisphaera mikurensis*	4	8	2	6	2	3	4	1	1	6	2	0	1	0
*Kuenenia stuttgartiensis*	3	0	0	0	0	24	0	0	0	0	0	0	0	0
*Chthoniobacter flavus*	23	5	3	8	1	0	0	0	1	14	6	0	2	0
*Verrucomicrobium spinosum*	13	12	0	5	2	0	0	0	3	11	7	1	0	1
*Lentisphaera araneosa*	23	35	0	7	3	0	1	10	13	74	50	0	1	0
*Zobellia galactanivorans*	22	8	2	19	0	0	0	20	8	15	16	2	2	1

Enzymes specifically involved in degradation of the *Porphyra* cell wall include the β-porphyranases in the GH16 subclass and the β-agarases that cleave β-1,4 glycosidic bonds (GH16, GH50, GH86, and GH118) [50]. Genes encoding members of these GH subclasses are unevenly distributed throughout the Planctomycetes. Putative orthologs for GH16 β-porphyranase genes, *porA*-*porE* (proteins characterized for *Z*. *galactanivorans*), are present in some characterized planctomycete genomes, but none encode a full set. *R*. *rubra* and *R*. *sallentina* each contain 3 β-porphyranase genes, one of which appears to be *porD*, while *R*. *maiorica* has only one ortholog. P3 and *P*. *mikurensis* each have one β-porphyranase gene, which clade with 72% node support (S5a Fig), while P1 and P2 have no β-porphyranase gene. Genes encoding GH16 β-agarases, such as those of *Z*. *galactanivorans* (*agaA*-*agaD*), are not present in P1, P2 or P3. Within the Planctomycetes, these genes are only in *R*. *sallentina* (1 gene) and *P*. *mikurensis* (2 genes); their phylogenetic placement in the context of four *Z*. *galactanivorans* β-agarase genes is presented in S5a Fig. There are, however, several Planctomycetes with GH50 and GH86 β-agarases, including P1, P2 and P3; GH118 β-agarases are not present in P1, P2 or P3.

GH117 α-neoagarobiases may be keystone enzymes for cleaving α-1,3 glycosidic linkages present in agarose [53]. Proteins of the GH43 subclass, which are structurally related to the GH117s [53], includes galactosidases, xylanases, arabinases and xylosidases, all of which would likely hydrolyze linkages in macroalgal cell walls. Furthermore, GH43 and GH117 proteins appear to be distantly related to the sulfatases based on the high incidence of GH43 and GH117 domain hits (BLASTp e-value < 10^−10^) within sulfatases of P1, P2 and P3.

The GH16 subclass includes genes encoding κ-carrageenases, which are found in P1 (IMG: 2643316630), *L*. *araneosa*, *Z*. *galactanivorans*, and the *Rhodopirellula*, including P2 (IMG: 2643292705). Genes putatively encoding ι-carrageenases are present only in *R*. *rubra* while λ-carrageenases are found in *R*. *rubra* and *R*. *sallentina*. While carrageenan is not present in *Porphyra umbilicalis* or any other member of the Bangiophyceae, it is the main cell wall polysaccharide of the red alga *Chondrus crispus*. In most areas of the North Atlantic, including Maine where P.um.1 was collected (S6 Fig), *P*. *umbilicalis* is positioned only 1–2 vertical meters from rich *Chondrus* beds.

Several of the investigated genomes also contain multiple genes encoding enzymes that potentially degrade fucans and alginates in brown algal cell walls. For instance, the GH29 (α-1,3/1,4-L-fucosidase) and GH95 (α-1,2-L-fucosidase) subclasses are highly expanded in 3 out of 6 members of *Rhodopirellula*, while the other three genomes, including P2, only contain 1 or 2 genes for these proteins (Table 2). The GH29 and GH95 subclasses have also expanded in P3, *L*. *araneosa*, and *Z*. *galactanivorans*. P1 and P2 contain multiple genes encoding PL6, PL9 and PL14 alginate lyases, while P3 has only a single gene member in PL6.

Some GH subclasses are represented by either zero or low membership in P1, P2 and P3. For example, P2 has no members in GH74, GH86 and GH118. There can also be major differences in the number of members of specific GH subclasses in the Planctomycetes [e.g., from 74 to 0 for GH43 and from 50 to 0 for GH117 (Table 2)]. Furthermore, 13 GH subclasses have maximum and minimum representations across the 6 *Rhodopirellula* genomes that differ by 10 or more members.

Cell wall polysaccharides comprise the majority of dry biomass of marine macroalgae, providing a rich carbon source for heterotrophic bacteria. Within meters of each other in the rocky intertidal and shallow subtidal zones of the North Atlantic shore are red algae with cell walls rich in carrageenan or agar rather than porphyran, brown algal kelps (subtidal) and rockweeds (high to low intertidal) that contain sulfated fucans and alginate, green macroalgae that have ulvans (sulfated glucuronoxylorhamnogalactans) and, especially in brown and green macroalgae, considerable cellulose [16]. It is unclear how much specificity there is in the cell-wall digesting capability of macroalgal-associated bacteria, but genomic analyses of their wall digesting capabilities may help explain their relative abundances on different groups of marine algae. Furthermore, substrate availability also impacts expression of the bacterial hydrolytic genes. When grown on the brown algal carbohydrate reserve laminarin, *Z*. *galactanivorans* expresses *porA* and *porB*, which encode enzymes that cleave neoporphyranobiose (L6S-G) in agar polymers [50]. However, when *Z*. *galactanivorans* is grown on a red alga with an agar-containing wall, the *agaA*, *agaB*, *agaC* and *agaD* genes are expressed, while a porphyran substrate elicits expression of *agaA*, *agaB*, *agaC*, *porC* and *porE* [50].

Variation in distribution of different GH categories among the three different planctomycete isolates raises the possibility that these bacteria have preferred niches [30] among the macroalgae. For example, P3 appears to be adapted to degrading brown algal cell walls based on the large number of fucosidases encoded in its genome; these have low representation in P1 and P2 (Table 2). P1 and P2 both appear well-equipped to live on both green and red algal cell walls based on their expanded arsenal of cellulases, arabinases, xylanases, agarases, porphyranases, galactanases, and carrageenases; GH10 xylanases comprise one of the largest expansions in P1 and P2 (Table 2).

## Horizontal Gene Transfer

Expansion of protein-coding gene families involving intra-chromosomal gene duplications (IGD) and horizontal gene transfers (HGT) is a key component of adaptive evolution. The relative impacts of IGD and HGT on bacterial evolution have been debated [42,54], with likely different roles in niche adaptation for paralogs acquired through IGD and xenologs acquired through HGT [55].

In general, definitive evidence for HGT is difficult to obtain; however, support can be acquired through various semi-quantitative metrics involving comparisons against “true” evolutionary lineages (as predicted in Fig 1). These metrics include (1) high bootstrap support for heterophyletic clades {except in cases of long-branch attraction [56]}, and (2) markedly higher sequence identity to gene(s) in more distantly related organisms than to orthologs in close relatives. Using such metrics, we predict numerous instances of HGT between the Planctomycetes and other bacterial/archaeal phyla and also between different genera within the Planctomycetes. Here we highlight cases of potential HGT in P1, P2 and P3 that appear to be associated with niche adaptations.

**Fig 5:**
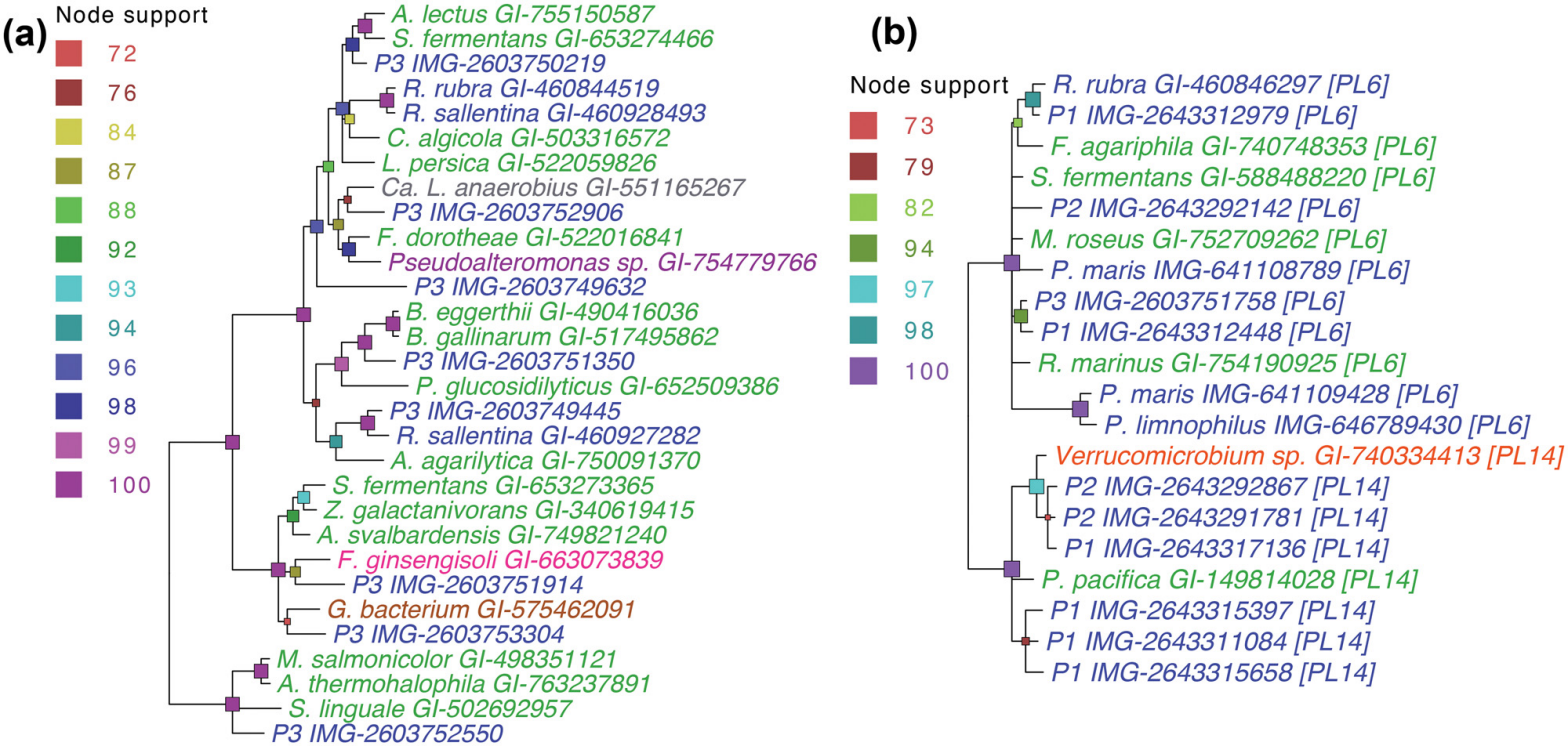
Phylogenies of polysaccharide-degrading enzymes indicate host adaptation. **(a)** Phylogeny of P3 α-L fucosidases (GH29). The genes included in the phylogeny are top hits having >50% sequence identity at 80% query coverage that were determined by BLASTp of each of the eight P3 fucosidases to the NCBI nr database and to the genomes included in this study. **(b)** Phylogeny of PL6 and PL14 alginate lyases. The genes included in the phylogeny are top hits having >50% sequence identity at 80% query coverage that was determined by the BLASTp of each of the P1, P2 and P3 alginate lyases to the NCBI nr database and to the genomes included in this study. In both **(a)** and **(b)**, genes are color-coded by organism as follows: Planctomycetes [blue], Bacteroidetes [green], Proteobacteria [purple], Verrucomicrobia [red-orange], Armatimonadetes [magenta], Gemmatimonadetes [brown], unclassified [gray]. Node support is from 1000 bootstrap iterations.

HGT of genes encoding polysaccharide-degrading enzymes can reflect adaptation to colonizing specific macroalgae. For instance, P1 appears to have acquired its ability to degrade κ-carrageenan from the Bacteroidetes; the P1 κ-carrageenase protein (IMG: 2643316630) clades with *Z*. *galactanivorans* and *C*. *drobachiensis* (98% node support) (S5b Fig), and is more similar in amino acid sequence to the protein of *Z*. *galactanivorans* (63% identity over 95% length) than to the closest planctomycete hit [*R*. *europaea* (GI: 460274492) at 44% identity]. Also, the phylogeny of eight α-L-fucosidases (GH29) in P3 is indicative of mixed evolutionary origins (Fig 5a). Only one of the eight fucosidases is terminally claded to another planctomycete (*R*. *sallentina*), while the others have their closest known relatives in Bacteroidetes, Armatimonadetes, and Gemmatimonadetes. Finally, both P1 and P2 show expansions in the family of PL14 alginate polysaccharide lyases, where a pair of P1 and P2 genes exhibits high amino acid sequence identity (74%), indicating a strong possibility for HGT of these genes (Fig 5b). HGT from free-living marine Bacteroidetes is known to have played a significant role in increasing degradative capability of marine Proteobacteria for digesting alginates [50] and for introducing genes encoding enzymes involved in alginate and porphyran digestion into human gut Bacteroidetes [26,50].

Genes in the planctomycete genomes potentially involved in adaptation to environmental stress are those most likely acquired by HGT. Multi-drug efflux pumps (pfam00873) are responsible for ejecting environmental and intracellular toxins such as metabolites, dyes, detergents, bile salts and antibiotics from cells. In *E*. *coli*, mutations in genes associated with TolC-dependent efflux systems cause up-regulation of various stress responses in *E*. *coli* [57]. P1 and P2 both contain a gene for an AcrB-type efflux pump, which is an inner membrane component of a TolC system. These P1 and P2 genes (IMG: P1–2643312425, P2–2643289582) encode proteins that are highly similar in sequence (83% identity over 99% of length) and do not appear to have vertically transmitted homologs in other Planctomycetes including *Rhodopirellula*, the genus to which P2 belongs (S7a Fig). The next closest match to the P2 protein is encoded by *R*. *maiorica*, at 52% sequence identity. These observations could reflect recent HGT between P1 and P2, or sequence convergence driven by purifying selection from shared environmental pressures reflecting variation in substrate specificities.

Amino acid transporters can be part of cellular stress response mechanisms, including those of the acid resistance system in *E*. *coli* [58], salt-stress induction of proline transporters in yeast [59], and the eukaryotic response to protein synthesis inhibition by oxidative stress [60]. A highly conserved amino acid transporter in P1 and P2 (66% amino acid identity over 99% of the length; IMG: P1–2643312291, P2–2643289856), but not encoded on any of the other planctomycete genomes, displays homology to transporters encoded on the genomes of a few members of the Bacteroidetes and Proteobacteria, and more broadly to various halophilic archaeal genomes (S7b Fig); these findings suggest the occurrence of HGT from Archaea to Bacteria, and then among a few bacterial phyla including the Planctomycetes. While the physiological role of this transporter is not known, it may function in response to frequent stresses in the intertidal zone, including high salinity and the absorption of excess excitation energy.

Genomic islands (GI) are horizontally transmitted gene clusters, generally mediated by transposable elements (TEs), that can facilitate adaptation to specific environments by conferring a selective advantage to the recipient [61]. P1, P2 and P3 contain putative GIs that span 4.2, 187.1 and 248.7 kbp, respectively. P3 has the largest number of TE-associated genes (Table 1) and also contains the largest total GI region (S8 Fig, S1 and S2 Texts). Functional predictions and the distributions of P1, P2 and P3 genes occurring in GIs are available in S3 Data. Notably, one of the P3 GH29 α-L-fucosidases (IMG: 2603749632) occurs in a GI. In addition, P1, P2 and P3 and many other Planctomycetes contain degenerate tRNA gene clusters with large numbers of partially degraded tRNAs, which are often acquired through HGT and thus, may be dispensable to the carrier organism [62,63]. Perhaps the most notable horizontal acquisition by the Planctomycetes is of a highly canonical isoleucine tRNA gene (tRNA-UAU) that occurs as a single-copy within degenerate tRNA gene clusters in several planctomycete genomes, including P1, P2 and P3. Codon usage analysis suggests that tRNA-UAU facilitates the translation of more recently acquired genes (such as genes in GIs), thereby increasing the rate at which new protein functions are established (S2 Text).

### Selenoproteins in P1 and P2

Adaptation to stress conditions has also been associated with selenoproteins, or enzymes containing selenocysteine (Sec) amino acid residues that generally confer increased catalytic efficiency compared to their sulfur-based, cysteine-containing homologs [64–66]. Most known selenoproteins have redox functions [67], and it has been suggested that the increased catalytic activities of selenoproteins are most beneficial in extreme environments associated with high levels of oxidative stress [68]. The largest known selenoproteome belongs to the harmful pelagophyte *Aureococcus anophagefferens* [69]. This picoplankton occurs in dense estuarine blooms where a portion of the cells are exposed to high light, elevated temperatures and osmotic stress [70]. Exposure to excessive light causes algae to produce reactive oxygen species, which must be quickly detoxified to avoid cellular damage [71].

**Fig 6:**
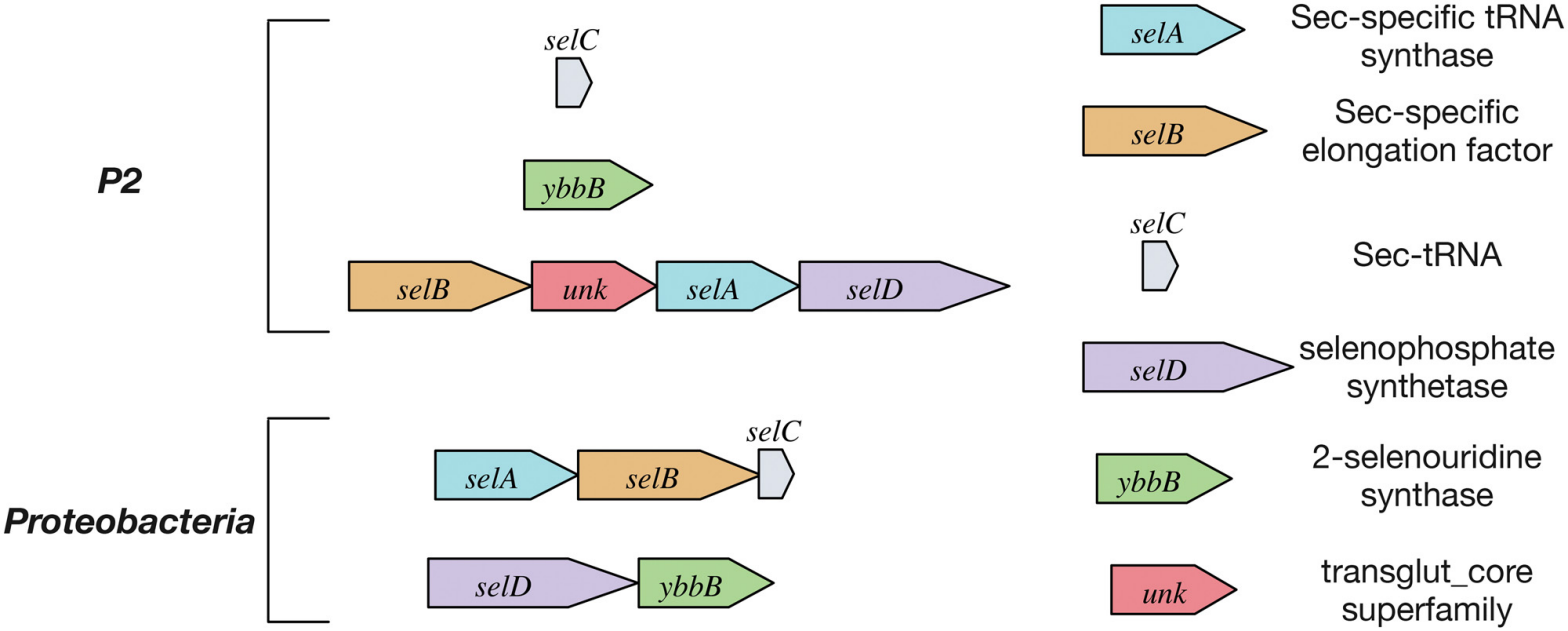
Comparison of operons encoding genes required for selenocysteine insertion and selenophosphate synthesis/utilization. For Proteobacteria, the two operons shown generally represent conserved structures for the majority of Sec-encoding and Sec-utilizing proteobacterial species. The Sec-insertion operon structure shown for P2 has not been found in other known genomes (NCBI), including P1. An additional gene is shown that contains a transglut_core domain (PFAM001841; likely to have cysteine protease function in prokaryotes).

Selenocysteines are co-translationally inserted into proteins by the selenosome complex [72], which requires 4 dedicated selenocysteine-associated genes (S8 Table). P1 and P2 both contain full sets of genes required for Sec-insertion during protein synthesis as well as genes for 2-selenouridine synthase (S8 Table), which improves base-pair discrimination in select tRNAs. P2 has an operon-like arrangement of these genes that is unusual in comparison to Sec-insertion operons in Proteobacteria (Fig 6), the phylum with the most known selenoproteomes (S2 Text); Sec-insertion genes in P1 are not co-localized. Also, Sec-insertion genes of both P1 and P2 appear to have mixed evolutionary origins (S2 Text). Two other planctomycetes, *G*. *obscuriglobus* and *I*. *pallida*, contain full sets of genes required for Sec-insertion, but neither of these genomes contain genes for 2-selenouridine synthase (S8 Table). We did not find genetic evidence for selenocysteine usage in P3.

**Fig 7:**
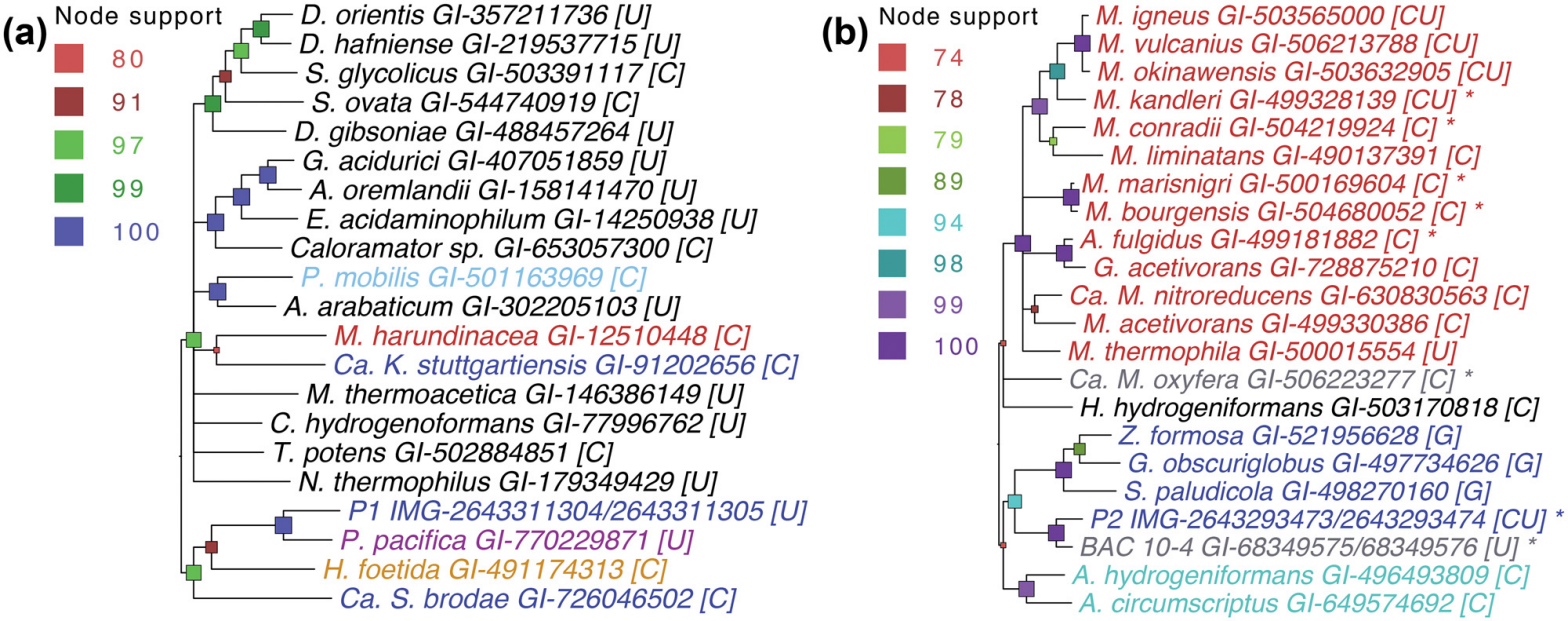
Horizontal gene transfer of selenoprotein genes reflects adaptation to stress conditions. **(a)** Phylogeny of formate dehydrogenase α subunit (*fdhA*). Closest non-redundant hits (BLASTp against NCBI nr) to the P1 selenoprotein sequence are shown. **(b)** Phylogeny of formylmethanofuran dehydrogenase β subunit (*fmdB*). Closest non-redundant hits to the P2 selenoprotein sequence are shown. Asterisk indicates a similar formylmethanofuran dehydrogenase operon structure as in P2 (*fmdD*-*fmdB*-*fmdA*-*fmdC*). In both **(a)** and **(b)**, genes are color-coded by organism as follows: Planctomycetes [blue], Proteobacteria [purple], Acidobacteria [orange], Thermotogae [light blue], Firmicutes [black], and Archaea [red], Synergistetes [cyan], unclassified [gray]. Sequences containing selenocysteine are marked with [U] and cysteine-containing sequences are marked with [C]. Node support is from 1000 bootstrap iterations.

Genes encoding putative selenoproteins in P1 and P2 were identified as described in the **Methods**, and are listed in S9 Table. In P1, a formate dehydrogenase α subunit (*fdhA*) is one of six putative selenoproteins with antioxidant activity. In Proteobacteria, *fdhA* is generally located near the Sec-insertion operon and may play a role in maintaining the Sec-insertion and decoding traits in bacteria [73]. In P1, the Sec-insertion genes and *fdhA* are not co-localized, but instead, *fdhA* forms an operon with *nuoEF*, genes that encode NADH:ubiquinone dehydrogenase I chains E and F (not selenoproteins). This P1 *fdhA* operon is well conserved (65% amino acid identity) in the myxobacterium *Plesiocystis pacifica* SIR-1 (a proteobacterium isolated from beach seagrass, *Zostera* sp.), but not in any other genome (in NCBI). Phylogenetic analysis indicates that the *fdhA* gene was part of multiple HGT events involving the Planctomycetes, including HGT between the P1 and *P*. *pacifica* lineages (Fig 7a).

Fig 7b shows a phylogeny of formylmethanofuran dehydrogenase β subunit (*fmdB* gene), which is encoded as a selenoprotein in P2, several Archaea, and two unclassified bacteria; glycine-containing homologs occur in three other Planctomycetes: *G*. *obscuriglobus*, *S*. *paludicola*, and *Z*. *formosa*. The closest match (71% amino acid identity) to P2 *fmdB* is on a fosmid associated with an uncultured bacterium from the freshwater lake, Lake Washington [74]. In P2 and the Lake Washington bacterium, *fmdB* is part of the *fmdD*-*fmdB*-*fmdA*-*fmdC* operon; this operon structure also occurs in several Archaea as well as Candidatus *Methylomirabilis oxyfera*. Some organisms, including P2, contain both selenocysteine and cysteine-forms of *fmdB*. In *M*. *kandleri*, these two forms are differentially expressed in response to selenium availability [75].

## Conclusion

This work has revealed numerous metabolic adaptations to the life style of planctomycete colonists of macroalgae within the intertidal zone, including the presence of large families of genes encoding sulfatases and hydrolases that degrade polysaccharides, multidrug transporters, and selenoproteins. Many of the hydrolytic enzymes allow P1, P2 and P3 to feed on the cell walls of the three major macroalgal groups (brown, green and red algae), but there are also suggestions of specialization for specific macroalgal hosts. Evidence for extensive HGT from the Bacteroidetes and Proteobacteria to the Planctomycetes emphasizes the intimate associations among these groups of bacteria on the macroalgal thallus. The interactions of the bacteria with each other, and with their associated macroalgae, are likely to reflect important physiological interactions that allow for the successful cohabitation of the bacteria and alga, and also offer the potential for genetic exchange that continually tailors bacteria to changing environmental conditions and macroalgal distributions.

## Supporting Information

S1 Data Gene family assignments for 22 marine bacterial species of the PVC superphylum and *Zobellia galactanivorans*. (XLSX)

S2 Data CAZY domain hits for 23 genomes. (XLSX)

S3 Data Predicted functions for genes found in genomic islands in P1, P2 and P3. (XLSX)

S1 Fig 16S rDNA phylogeny of three novel Planctomycetes and related species. Consensus maximum likelihood trees from 1000 bootstrap iterations are shown. Internal nodes are color-coded (indicated to the left of each tree) based on bootstrap support values. Taxa are color-coded by habitat: marine (blue), freshwater (orange), marine/brackish (purple), soil (green).
(TIFF)

S2 Fig Circular maps showing degree of genomic synteny between species of the genus *Rhodopirellula*. **(a)** Synteny between *R*. *baltica* and *R*. *europaea*. **(b)** Synteny between *R*. *baltica* and P2. In both **(a)** and **(b)**, the outer circle serves as a template genome (*R*. *baltica*) with all protein-coding genes represented as individual lines along the circle. The arrangement of genes in the outer circle preserves actual gene order and genomic distances to scale. On the inner circle, protein-coding genes from a secondary genome (*R*. *europaea* in **(a)** and P2 in **(b)**) are aligned to the template genome; each individual gene is placed next to its best BLASTp hit in the template genome. In both outer and inner circles, genes are colored based on genomic position using a continuous RGB color scale from red to blue; thus, similarly colored genes that are in the same circle occur nearby in the genome.
(TIFF)

S3 Fig Correlation between genome size and the number of genes in families for marine bacteria. The number of genes in families was determined using a network-based approach as described in S1 Text. The blue line represents a best-fit line, and the darker shaded area shows the 95% confidence interval.
(TIFF)

S4 Fig Distribution of gene functions for gene families containing singletons, or with 2–5, or 6+ members in P1, P2 and P3. The x-axis gives various COG functional categories represented by different colors and letters, as defined in Fig 2b. The numbers of genes/domain hits that correspond to each functional category can be found on the y-axis.
(TIFF)

S5 Fig Phylogeny of β-porphyranases, β-agarases and κ-carrageenases. **(a)** Phylogeny of β-porphyranases and β-agarases in the Planctomycetes. P1 and P2 do not have orthologous genes encoding β-porphyranases and β-agarases characterized in *Z*. *galactanivorans*. **(b)** Phylogeny of κ-carrageenases. P1 and P2 each have one copy of the κ -carrageenase gene as shown. In both P1 and P2, it is unlikely that these genes were vertically transmitted. In both **(a)** and **(b)**, leaves are color-coded by phylum as follows: Planctomycetes [blue], Bacteroidetes [green], Proteobacteria [purple], Verrucomicrobia [red-orange]. Node support is shown from 1000 bootstrap iterations.
(TIFF)

S6 Fig The intertidal zone at Schoodic Point, Maine, where P.um.1 was isolated. **(a)** *Porphyra umbilicalis* (arrow) is present in abundance and localized amidst large expanses of green and brown algae at low tide. **(b)** Close-up of *Porphyra umbilicalis* growing, typically, centimeters from macroalgae belonging to the other major groups, such as the large brown rockweed *Fucus vesiculosus* and green algal mats of *Ulothrix*/*Urospora* (Chlorophyta).
(TIFF)

S7 Fig Phylogeny of amino acid transporters and multi-drug efflux pumps. **(a)** Phylogeny of amino acid transporters that likely originated in halophillic Archaea. A few species from Proteobacteria [purple], Bacteroidetes [green], and Planctomycetes [blue] are shown to have acquired this gene. **(b)** Phylogeny of multidrug efflux pumps that are highly conserved in P1 and P2. The closest planctomycete matches are included in the phylogeny. The P1 and P2 efflux pumps share 83% amino acid sequence identity, and display ~50% sequence identity with the other encoded proteins used in this analysis. Node support is shown from 1000 bootstrap iterations.
(TIFF)

S8 Fig Genome maps showing the positions of genomic islands (GI). Circular diagrams represent the genome of P1, P2 and P3, as labeled. Genomic islands are marked by colored patches that span various parts of a genome. GI predictions by SIGI-HMM, which makes predictions based on codon usage, are shown in orange. Predictions by IslandPath-DIMOB, which makes predictions based on abnormal sequence composition and the presence of mobile genetic elements, are shown in blue. Red patches integrate both SIGI-HMM and IslandPath-DIMOB predictions. Circular diagrams were generated using IslandViewer2 (www.pathogenomics.sfu.ca/islandviewer).
(TIFF)

S1 Table Housekeeping genes used for phylogenetic analyses. (XLSX)

S2 Table Strain and assembly information for genomes used in this study. (XLSX)

S3 Table Distribution of tRNA genes across isotypes. (XLSX)

S4 Table 16S rDNA sequence identities between selected planctomycete genomes. (XLSX)

S5 Table Summary statistics of genes in gene families. (XLSX)

S6 Table Highly represented gene families in Planctomycetes. Abbreviated family names are response regulators (RR), serine/threonine protein kinases (STPK), domain of unknown function (DUF), ABC transporters (ABC), glycosyltransferases (GT1, GT2), NADH-dependent dehydrogenases (NDDH), membrane-bound dehydrogenases (MBDH).
(XLSX)

S7 Table Selected CAZY glycoside hydrolase and polysaccharide lyase domains. The GH and PL domains below are present in abundance, expanded, or overrepresented in P1, P2, or P3.
(XLSX)

S8 Table Genes involved in selenocysteine insertion and utilization in Planctomycetes. IMG gene identifiers are provided.
(XLSX)

S9 Table Selenoprotein gene candidates in P1 and P2. (XLSX)

S1 Text Supplementary Methods. A more detailed description of our methods including sample preparation, sequencing and assembly, sequencing error correction, genome annotation, phylogenetic analyses, analysis of gene families, analyses of sulfatases and CAZymes, and detection of selenoproteins.
(DOCX)

S2 Text Supplementary Results. Additional results that supplement the findings presented in the main article are provided here.
(DOCX)
