# Supplementary figures and images for: Genome Analysis of Planctomycetes Inhabiting Blades of the Red Alga Porphyra umbilicalis
Source: PLoS One. 2016 Mar 25;11(3):e0151883. doi: 10.1371/journal.pone.0151883 (PMC4807772; doi:10.1371/journal.pone.0151883)

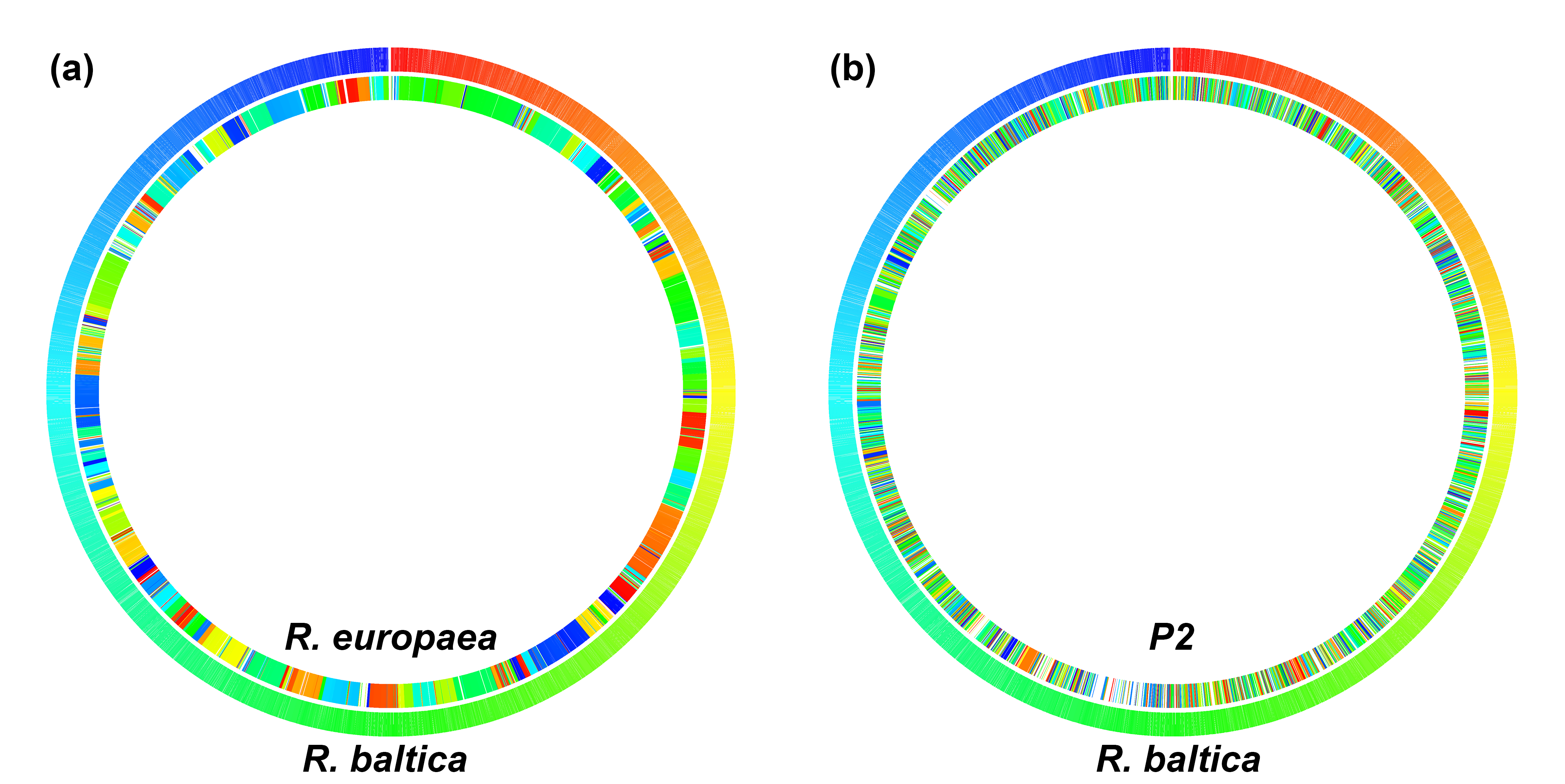

Supplement: S2 Fig — (a) Synteny between R. baltica and R. europaea. (b) Synteny between R. baltica and P2. In both (a) and (b), the outer circle serves as a template genome (R. baltica) with all protein-coding genes represented as individual lines along the circle. The arrangement of genes in the outer circle preserves actual gene order and genomic distances to scale. On the inner circle, protein-coding genes from a secondary genome (R. europaea in (a) and P2 in (b)) are aligned to the template genome; each individual gene is placed next to its best BLASTp hit in the template genome. In both outer and inner circles, genes are colored based on genomic position using a continuous RGB color scale from red to blue; thus, similarly colored genes that are in the same circle occur nearby in the genome. (TIFF) [file pone.0151883.s005.tiff]

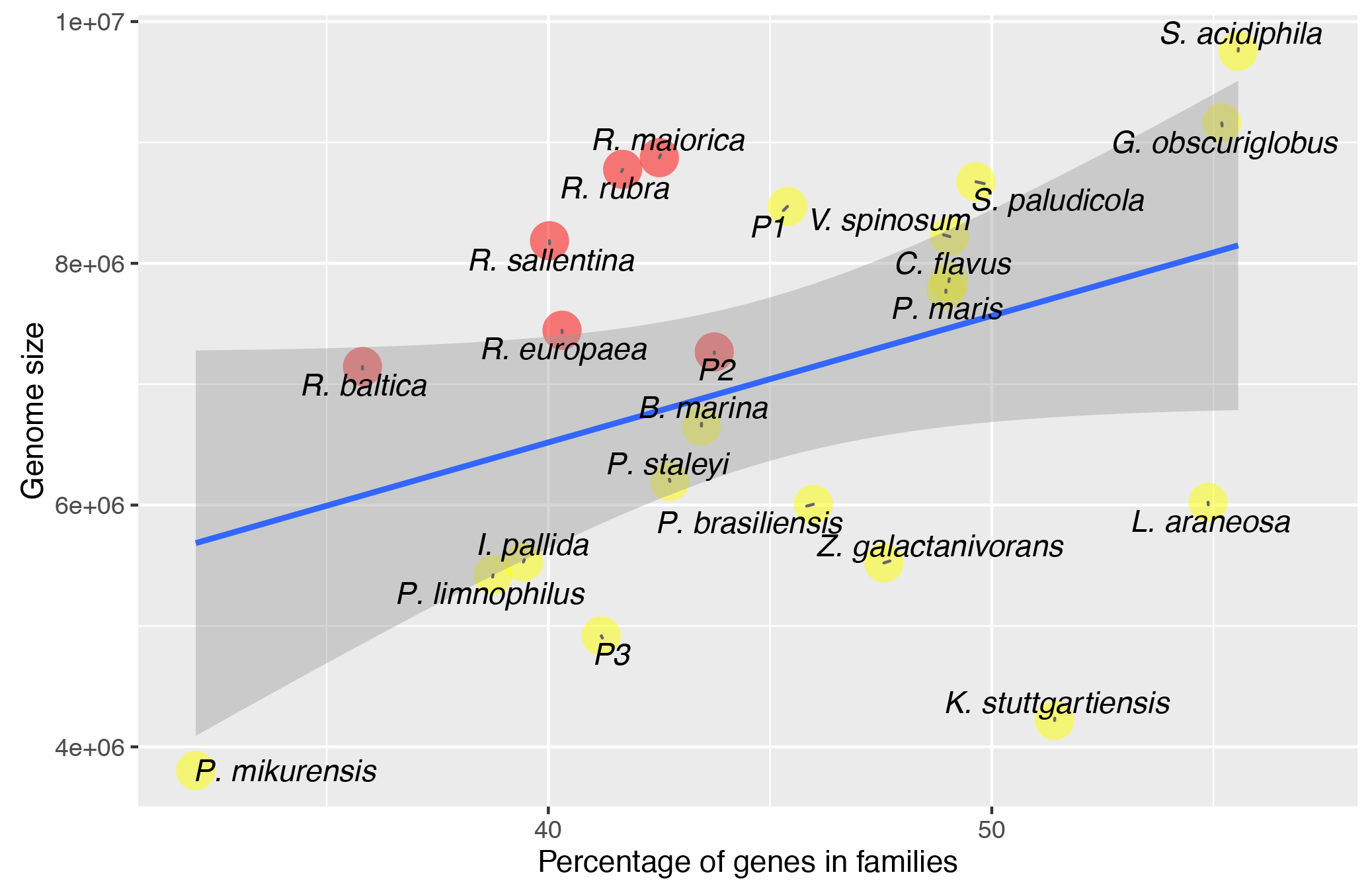

Supplement: S3 Fig — The number of genes in families was determined using a network-based approach as described in S1 Text. The blue line represents a best-fit line, and the darker shaded area shows the 95% confidence interval. (TIFF) [file pone.0151883.s006.tiff]

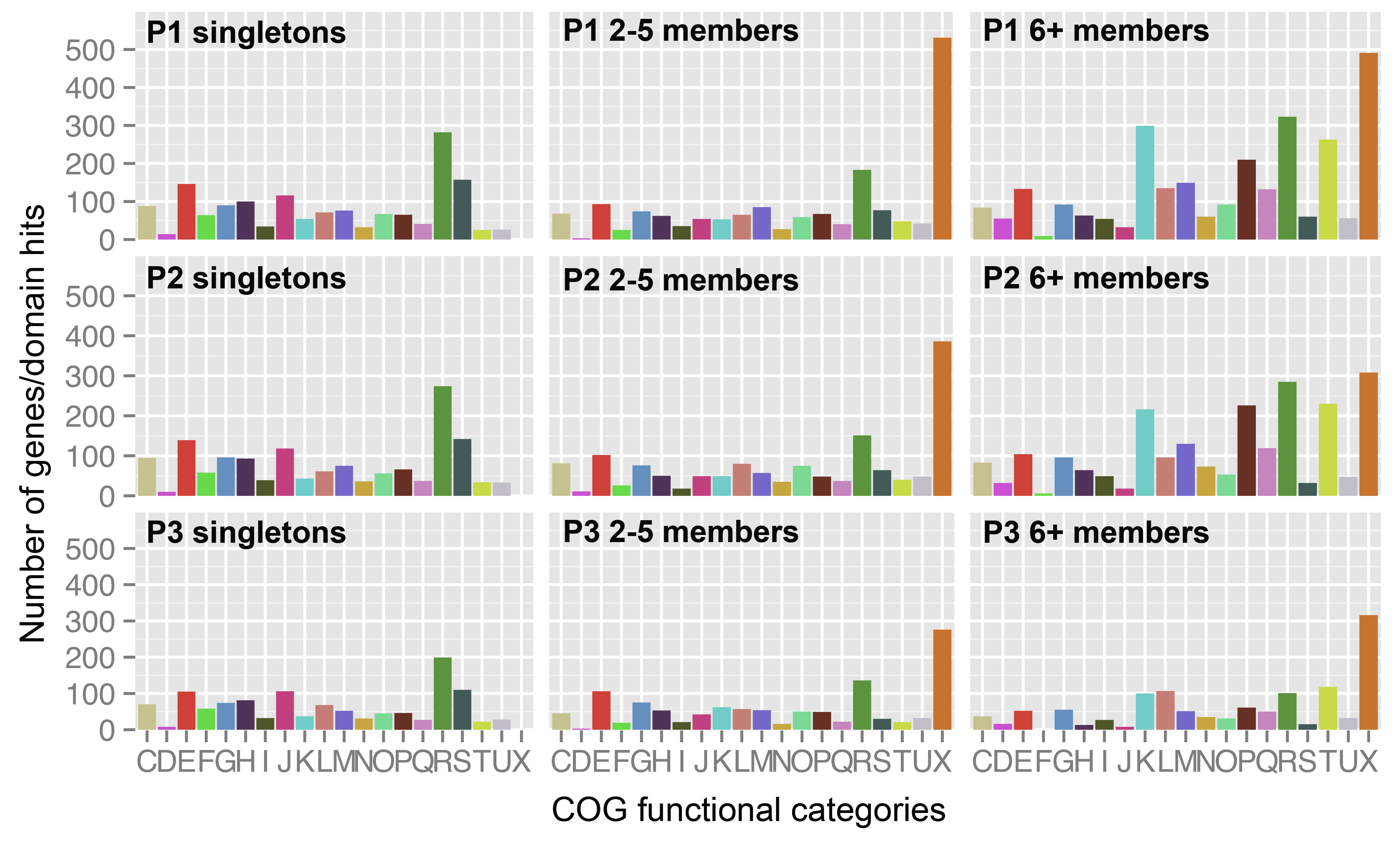

Supplement: S4 Fig — The x-axis gives various COG functional categories represented by different colors and letters, as defined in Fig 2b. The numbers of genes/domain hits that correspond to each functional category can be found on the y-axis. (TIFF) [file pone.0151883.s007.tiff]

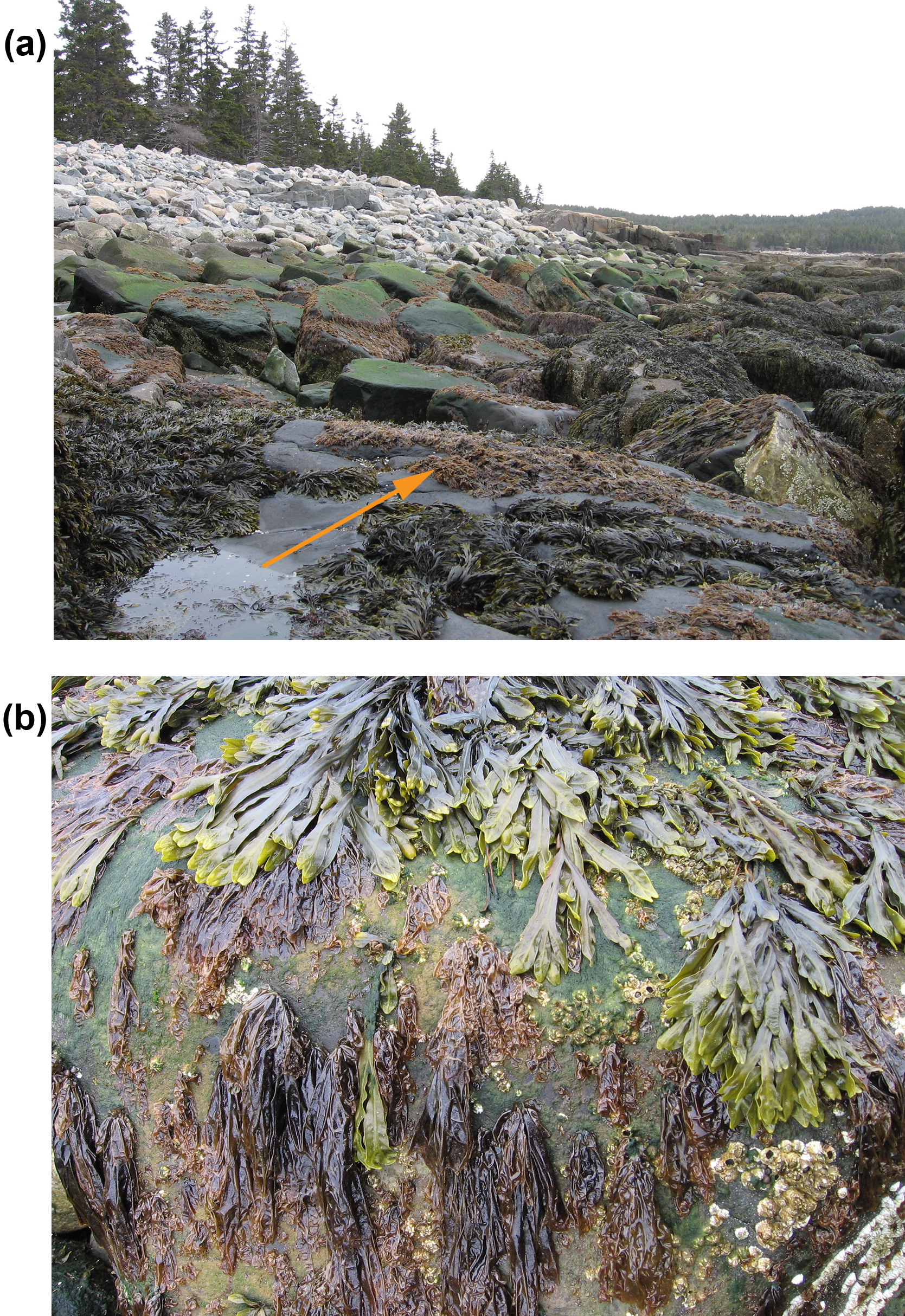

Supplement: S6 Fig — (a) Porphyra umbilicalis (arrow) is present in abundance and localized amidst large expanses of green and brown algae at low tide. (b) Close-up of Porphyra umbilicalis growing, typically, centimeters from macroalgae belonging to the other major groups, such as the large brown rockweed Fucus vesiculosus and green algal mats of Ulothrix/Urospora (Chlorophyta). (TIFF) [file pone.0151883.s009.tiff]
