# Supplementary material for: Genome Analysis of Planctomycetes Inhabiting Blades of the Red Alga Porphyra umbilicalis
Source: PLoS One. 2016 Mar 25;11(3):e0151883. doi: 10.1371/journal.pone.0151883 (PMC4807772; doi:10.1371/journal.pone.0151883)
Supplement: S1 Text — A more detailed description of our methods including sample preparation, sequencing and assembly, sequencing error correction, genome annotation, phylogenetic analyses, analysis of gene families, analyses of sulfatases and CAZymes, and detection of selenoproteins. (DOCX) [file pone.0151883.s021.docx]

**Supplementary Methods**

Genome Analysis of Planctomycetes Inhabiting Blades of the Red Alga *Porphyra umbilicalis*

JW Kim, SH Brawley, S Prochnik, M Chovatia, J Grimwood, J Jenkins, K LaButti, K Mavromatis, M Nolan, M Zane, J Schmutz, JW Stiller, AR Grossman

**1. *Sample isolation and preparation***: Genetically clonal blades of P.um.1 were grown in West-McBride’s adaptation of Provasoli’s Enriched Seawater [[1](#_ENREF_1)] on a 14:10 (L:D) photoperiod at a constant light intensity of 60 μmol photons m^-2^ s^-1^ (Osram Sylvania 20 W cool white fluorescent tubes) and at 10°C. The original isolate was collected at Schoodic Point, Maine (44°20’1.68” N; 68°3’29.14”W) on April 3, 2008 [[2](#_ENREF_2),[3](#_ENREF_3)]. The clonal daughter blades from which DNA samples were isolated contained undescribed Planctomycetes and were grown up through 2-12 generations in the laboratory with weekly medium changes, and were treated several times for 6 d periods with penicillin (100 mg/l) and streptomycin (25 mg/l), including immediately before harvest of the material. Blades were frozen in liquid nitrogen and stored at -80°C.

DNA was extracted from 1-3 g of frozen tissue that was powdered in a mortar and pestle and then suspended in 20 mL Carlson lysis buffer containing 2% CTAB (see Qiagen QG07.doc Sept-01) and purified using the Qiagen Genomic-tip 500/G kit, as described by the manufacturer. A high %GC DNA band was isolated from purified DNA by ultracentrifugation in a CsCl gradient (1 g CsCl/ml in 10 mM Tris, pH 8.0, 1 mM EDTA, 1.3 µg/ml ethidium bromide); the centrifugation was overnight (12-14 h at 300,000 x *g* in a VTi65 rotor). Prior to sequencing, the high % GC DNA band was collected from the gradient, extracted 2x with 1 vol CsCl-saturated isopropanol and then precipitated overnight with 2 vol of 100% ethanol at RT. The precipitated DNA was pelleted by centrifugation (7,500 x *g* for 50 min), washed with 70% ethanol, dried for 15 min at RT and resuspended in 100 uL TE for 2 h at 50°C.

**2. *454 genomic library preparation, sequencing, and assembly***: A standard genomic DNA library was prepared for 454 sequencing. Briefly the procedure involved shearing 3 µg of gDNA by nebulization (N_2_ at 30 psi for 1 min) in buffer supplied with the Titanium library preparation kit (Roche, stock no. 04 852 265 001). The sheared fragments were purified using a Qiagen MinElute column, size-fractionated by agarose gel electrophoresis and fragments in the size range of 500-800 bp were eluted from the gel and purified using a QiaQuick column. The DNA size and quality were determined using the Agilent Bioanalyzer. Fragments were end polished with T4 polymerase from the Titanium library preparation kit (Roche, stock no. 04 852 265 001), purified using AMPure beads, and then ligated to 454 Titanium adaptors. Excess adaptors were removed using AMPure beads and the adaptor-modified double-stranded DNA was then denatured with alkali and the ssDNA templates isolated. The Bioanalyzer and RNA Pico 6000 chip were used to evaluate size and quantity of the ssDNA, which was then used for emulsion PCR (emPCR) followed by paired-end sequencing on the 454 platform.

To prepare the long distance 10 kb library, 15 µg of gDNA was sheared to 1 kb or larger using HydroShear (Digilab), the fragments were biotinylated, ligated to loxP adaptors and circularized through recombination by a Cre excision reaction. The circular DNA templates were randomly fragmented by nebulization, and then captured by streptavidin-coated magnetic beads followed by ligation of purified fragments to adaptors, which were then amplified by PCR using a primer set that annealed to the adaptors, one of which was biotinylated. Following AMPure (SPRI) bead size exclusion and streptavidin magnetic bead immobilization of dsDNA amplicons, ssDNA templates were isolated by alkaline treatment. The resulting ssDNA paired-end library was used for emPCR and subsequent sequencing with the 454 platform.

A preliminary 454 assembly was generated with Newbler (v.2.3-PreRelease-10/20/2009, Roche). Three scaffolds were much longer than the others and were identified as microbial by blasting their sequences to the nonredundant (nr) database at NCBI. These three scaffolds had lengths of 8.5, 7.3 and 3.8 Mbp. We established a procedure for assembly improvement which eliminated contigs that aligned to more than one of the three scaffolds; such sequences would interfere with correct scaffold generation. We also removed reads <80 bp and those with BLAST hits to *Porphyra* mitochondrial or chloroplast sequences. The 8.5, 7.3 and 3.8 Mbp scaffolds were then assembled individually with Newbler. We closed gaps to improve all three assemblies and completed their partial and/or fragmented 16S sequence so that a taxonomic analysis could be performed.

**3. *Additional Illumina sequencing, assembly and 454 homopolymer error correction***: Potential 454 homopolymer errors in the 8.5 and 7.3 Mbp scaffolds were corrected using previous obtained Illumina sequencing libraries (NCBI Sequence Read Archive libraries SRX116368 and SRX059849 from PRJNA234409). Illumina reads from the two libraries were aligned to the 8.5 and 7.3 Mbp scaffolds using BWA-MEM [4] and consensus sequences corresponding to each scaffold were obtained using nesoni-v0.132.

The 3.8 Mbp genome appeared to be incomplete based on its gene complement. We performed additional sequencing using Illumina MISeq/HISeq machines and obtained a 26.62 Gb sequencing read library that included 2×250 paired-end reads with varying insert sizes (400 bp and 800 bp) and 2×300 mate-pair libraries. We then removed reads corresponding to mitochondria, chloroplast, PhiX control library, and simple repeats. This Illumina read library was assembled using ALLPATHS-LG [[5](#_ENREF_4)], resulting in 18,748 scaffolds. Here, we found a 4.9 Mbp scaffold that corresponded to the 3.8 Mbp Newbler scaffold obtained via earlier 454 sequencing.

The three largest microbial scaffolds assembled from all sequencing runs (454 and Illumina) correspond to the genomes of Planctomycetes that we have designated P1 (8.5 Mbp), P2 (7.3 Mbp) and P3 (4.9 Mbp). An iterative local assembly extension strategy was implemented using Velvet v1.2.10 [[6](#_ENREF_5)]. Gaps in specific genes (rDNA and housekeeping genes used for phylogenetic analysis) were closed by iteratively extending the assembly inward using subsets of 454 reads that partially align at high sequence similarity (≥99%) to the sequenced regions flanking the gaps, while also extending into gaps. After closing gaps, we defined boundaries of rDNA sequences using the *R. baltica* 16S rDNA as a reference template.

Because of some ambiguities in the P3 16S DNA sequences, we used the purified high GC-band (CsCl preparation) extracted from P.um.1 to recover and verify the bioinformatic assembly of P3 16S rDNA. An initial PCR reaction with primers P3-F2 and P3-R2 (98°C, 30 s; followed by 35 cycles of 98°C, 10 s; 71°C, 30 s; 72^o^C, 45 s; with a final extension step at 72°C for 5 min) was followed by a nested PCR using the diluted FP3-2/P3-R2 PCR product and P3-F3/P3-R3 primers (same PCR program as for F2/R2) and PLA-46-F2 and UEB-1392R primers (98°C, 30 s; 35 cycles of 98°C, 10 s; 58°C, 30 s; 72°C, 45 s; with a final extension step at 72°C, 5 min) in separate reactions. The following primers were used for these procedures: P3-F2 - ACCCTCGCGACTTTGTTCGC, P3-R2 – TGGTGAATTGTGCTTCGTCGCG, P3-F3 – TTGGTGATGGTGGTGGAGGCAC, P3-R3 – GCGAACGACTTAGCGGACCAAC, PLA-46-F2 – GGATTAGGCATGCAAGTC, UEB-1392R – ACGGGCGGTGTGTAC. P3-primers were designed with Primer3Plus (http://www.bioinformatics.nl/cgi-bin/primer3 plus/pimer3plus.cgi) against contigs expected to fill gap regions of the 16S rDNA. PLA-46-F2 and UEB-1392R are universal planctomycete and eubacterial primers, respectively. PCR reactions were performed in 50 μl, with each reaction containing 0.5 μM of each primer, 1 U of NEB’s Phusion® High Fidelity DNA Polymerase in the high GC buffer supplied with the enzyme, and 200 μM of each dNTP. The P3-F2/P3-R2 reaction contained 60 ng of the P.um.1 DNA; the P3-F2/P3-R2 PCR product was diluted 1:500 in nuclease-free water and added at 1 μl/reaction to the nested P3-F3/P3-R3 PCR or as a 1:200 dilution (4 μl/reaction) with the PLA-46-F2 and UEB-1392R primers. PCR products were cleaned with a QIA-quick PCR clean-up kit (Qiagen) and sequenced by Elim Biopharm ([www.elimbio.com](http://www.elimbio.com)).

**4. *Genome annotation***: The protein coding genes of the P1, P2 and P3 genomes were analyzed using the Integrated Microbial Genomes (IMG) tool (http://img.jgi.doe.gov/) and classification tools provided in the Clusters of Orthologous Genes [[7](#_ENREF_6)] database [[8](#_ENREF_7)]. Annotation was also based on searches of sequence similarity to proteins in the NCBI and UniProt databases. Simple tandem repeat content was estimated using TandemRepeatsFinder v4.04 [[9](#_ENREF_8)] with the following parameters: match = 2, mismatch = 5, indel = 7, min score = 25, max period = 2000. The CRISPRFinder web service [[10](#_ENREF_9)] was used to identify putative CRISPRs. Predictions for tRNA genes were generated using tRNAscan-SE v1.31 [[11](#_ENREF_10)]. The numbers of rRNA and other non-coding RNA gene predictions were gathered from the IMG database. We also scanned for transposable element (TE)-associated genes, which were predicted based on the presence of TE-associated PFAM domains [[12](#_ENREF_12)].

**5. *Phylogenetic analyses***: We generated an initial phylogeny based on 16S rDNA sequences for 25 bacterial species using RAxML [[13](#_ENREF_14)] with the GTR-GAMMA model. We then built a statistically robust phylogeny by sampling across multiple protein-coding loci [[14](#_ENREF_15)] to identify highly conserved protein-coding genes present in single copy in each of the 23 genomes used in this study. To achieve this, we gathered an initial set of 47 housekeeping genes [[15](#_ENREF_16),[16](#_ENREF_17)] that are unlikely candidates for lateral gene transfer and are well-conserved across all domains of the tree of life. From the initial set, 8 genes (*abc*, *asnS*, *cysNC*, *fliI*, *fusA*, *glnS*, *groL*, *pgk*) were removed because of ambiguous orthology/paralogy relationships. The remaining 39 genes comprising our “core” gene set are listed in **S1 Table** with their encoded functions.

Prior to alignment and tree generation, we analyzed the quality of individual genes in the core set. First, we aligned orthologs from each genome using the L-INS-i executable in the MAFFT package [[17](#_ENREF_18)]. We then manually inspected the resulting alignments for missing regions, large-scale misalignments, and start and stop codons. In some cases the initiator codon at the 5’-end of a gene was likely not correctly chosen in the generation of the gene model, and therefore it was necessary to extend the gene model to an alternate start codon in order to more faithfully capture the encoded full-length protein as determined by sequence alignments against related genes. Multiple alignments for the 39 core genes were trimmed to remove gaps using trimAl [[18](#_ENREF_19)] and then concatenated, adhering to a predetermined, randomized gene order. A maximum-likelihood phylogeny based on 8,725 amino acid positions was inferred using RAxML with an optimal set of parameters for tree-generation estimated in ProtTest3 [[19](#_ENREF_20)]. The final consensus tree was generated from 1,000 bootstrap iterations. We used resources at the CIPRES Science Gateway [20] for intensive phylogeny computations.

**6. *Analysis of gene families***: Protein-coding gene families were defined by comparing all 136,494 protein-coding gene sequences across the 23 genomes listed in **S2 Table**. We implemented a network-based approach that detects paralogous gene-families present within a genome as well as congruent gene families across pairs of genomes. Our motivation for developing this approach was (1) to avoid setting hard sequence similarity thresholds for determining gene-family boundaries, and (2) to improve upon the reciprocal best BLAST hit method for detecting homologous genes across genomes, which is ill-suited for comparing moderately to highly diverged genomes containing many instances of gene loss, duplication, and horizontal transfer [[21](#_ENREF_22),[22](#_ENREF_23)].

Our method considers both network topology and sequence similarity for assigning genes to specific families and detecting congruent gene families among the selected organisms. This approach relies on Infomap [[23](#_ENREF_24)], an algorithm that identifies communities in large networks by finding the partitioning that minimizes the information necessary to describe a random walker’s movements on a network. Our input network to Infomap consisted of vertices representing all protein-coding genes in a genome with edges representing the degree of homology between any two vertices, or pair-wise sequence similarity. For quantifying the strengths of pairwise relationships, we used the Neighborhood Correlation (NC) method, which identifies homologous protein-coding genes by comparing the topology of sequence similarity networks. The main advantage of NC over traditional pair-wise sequence similarity metrics such as BLASTp is that it robustly captures relationships between multi-domain proteins.

NC scores for all pair-wise (136,494 by 136,494) comparisons were generated using the NC standalone script (v2.1) obtained from http://www.neighborhoodcorrelation.org/. The magnitude of the score provides a measure of homology between two protein-coding genes. To generate the score we first established all-by-all BLASTp bit score matrices (symmetrical) for each genome under study. BLASTp was executed using the BLASTALL script from the NCBI-BLAST package (ftp://ftp.ncbi.nlm.nih.gov/blast/executables/release/LATEST/) with the following parameters as specified in the NC v2.1 README text: -p blastp –e [10 * number of sequences] –Y [(# residues in dataset)^2^]. We investigated higher-level relationships by generating networks where vertices are encoded protein sequences with edges that are given weights corresponding to NC scores. Two protein sequences were assigned an edge between them only if the NC score for their comparison was greater than a predetermined threshold of 0.4. We chose this threshold based on previous findings [[24](#_ENREF_25)] that examined the distribution of NC scores from the comparison of mouse and human encoded proteins, and found that an NC score threshold of 0.4 gave low false positives and reasonable numbers of false negatives. Gene families were defined based on the network sub-communities detected by Infomap.

We initially classified gene-families by transferring the annotations for individual genes (based on encoded proteins) from original genome sequencing projects. In cases of conflicting annotations, or when gene families encoded mostly hypothetical proteins, we referenced sequence similarity searches in the Conserved Domains Database [[25](#_ENREF_26)]. Comparisons of gene-families across genomes can be problematic when a genome contains multiple gene-families with the same annotations (e.g., families encoding hypothetical proteins, transposases, or RNA polymerase sigma factors). To address this issue, we implemented a two-step graph-building strategy for gene-family comparisons at the genome level. Given two genomes for comparison, our goal was to determine one-to-one assignments between congruent gene-families from different genomes so that we could infer homology between matched families, and infer gain and loss in cases of non-matches. The problem above is analogous to finding a maximum matching in a weighted bipartite graph where genomes *A* and *B* are represented as two disjoint sets of vertices (each vertex represents a gene-family), and each edge must connect a vertex in *A* to a vertex in *B* such that each vertex has at most one connection. Given that edge weights quantify the degree of congruency between vertices, the maximum condition requires that the sum of the edge weights in the final matching have a maximal value. To ensure that a maximal matching between two genomes makes biological sense, edge weights must accurately capture the degree of relatedness between gene-families. Our edge weights were derived from a score based on two criteria: (1) the number of orthologous pairs between gene-families in different organisms, and (2) the degree of sequence similarity between each ortholog pair within the family. In order to implement such a score, it is necessary to assign orthologous and paralogous relationships between all genes being considered. We generated these assignments using a greedy strategy. Let *G*(*V*,*E*) be a graph where *V* is the set of vertices representing all genes in the two gene-families and *E* is the set of edges between them with NC scores as edge weights. Starting with the set of unconnected vertices (no edges included), we add the edge with the highest NC score first, and then consider the remaining edges in order of their decreasing NC scores. For each remaining edge under consideration, if both of the vertices defining the edge already have one or more connections, then we discard that edge and move on to the next one. This process is iterated until all edges have been considered. In the resulting graph, we initially label all edges connecting two members of the same gene-family as paralogs, and all edges connecting members of different gene-families as orthologs. This intermediate configuration allows multiple orthologous edges to associate with a single vertex. However, to resolve such cases, we only keep the orthologous edge with the highest NC score and reconfigure the remaining edges to be paralogous edges incident on the in-family vertex corresponding to the orthologous edge with the highest NC score. Finally, we sum NC scores across all remaining orthologous edges to generate a score to serve as edge weights for the maximum weighted bipartite matching phase.

**7. *Sulfatase classification and analysis***: Sulfatase subclasses were determined based on high-level clades in a maximum likelihood phylogeny of all sulfatase protein sequences for a given organism. Each resolvable clade was annotated as iduronate-2-sulfatase, heparan-N-sulfatase, mucin-desulfating sulfatase or choline sulfatase, based on BLASTp similarity with sequence in the UNIPROT TREMBL database. Most sulfatase sequences were assigned to high-level clades that were not clearly resolvable (low bootstrap support), or contained ambiguous electronic annotations; these “unclassified” sulfatases could be placed in the more general categories ‘arylsulfatase A’ and ‘galactosamine-N-acetyl-6-sulfatases’ (GALNS). We inferred the extracellular localization of sulfatases from the presence of a signal peptide as predicted by SignalP-4.1 [[26](#_ENREF_27)].

**8. *Comparative analysis of carbohydrate active enzymes***: The CAZY database contains sequences of enzymes that degrade, modify or create glycosidic bonds, with a description of CAZY classes given at http://www.cazy.org. The CAZY classes are GHs, glycosyltransferases (GT), polysaccharide lyases [[27](#_ENREF_28)], carbohydrate esterases (CE), and carbohydrate binding modules (CBM). We identified CAZYs in the 23 genomes used in our analyses with the CAZY Analysis Toolkit, which executes a BLASTp search against the CAZY database. All hits were gathered with the relatively stringent e-value of <10^-10^ for downstream analyses. All CAZY hits for 23 genomes are provided in **S2 Data**.

**9. *Identification of genes encoding selenoproteins and Sec insertion and utilization elements*:** The protein-coding Sec-insertion genes *selA*, *selB*, *selD,* and the 2-selenouridine synthase gene (*ybbB*) were identified by sequence alignments (BLASTp) against known bacterial homologs. Selenoprotein genes are generally overlooked during automated gene annotation since ‘UGA’, which codes for both selenocysteine and the opal stop, is an ambiguous codon in genomes containing selenoprotein genes. In addition to the occurrence of an in-frame ‘UGA’ codon, identification of selenoprotein genes requires additional verification, which can be achieved through homology searches (i.e. homologs that have a Sec residue at the analogous ‘UGA’ position) or detection of a selenocysteine insertion sequence (SECIS element) that occurs directly downstream of a selenocysteine ‘UGA’. The SECIS element adopts a hairpin-loop structure that interacts with the Sec insertion machinery, and is necessary for Sec insertion at the upstream ‘UGA’. Initial candidates for selenoproteins were identified based on the presence of in-frame ‘UGA’ codons. These candidate proteins were further checked for the existence of homologous protein sequences in other bacteria (in NCBI nr) that contain a Sec UGA codon at the same position; we also considered cases where homologs contained cysteine or glycine residues. Selenocycsteine insertion sequences (SECIS), which also help confirm identification of Sec proteins, were detected using an automated SECIS element identification algorithm called bSECISearch (http://genomics.unl.edu/bSECISearch/).

**References for Supplementary Methods**

1. Anderson RA (2005) Algal Culturing Techniques. Amsterdam: Elsevier. 578 p.

2. Blouin NA (2010) Asexual reproduction in *Porphyra umilicalis* Kutzing and its development for use in mariculture: University of Maine. 151 p.

3. Blouin NA, Brawley SH (2012) An AFLP analysis of clonality in widespread asexual populations of *Porphyra umbilicalis* (Rhodophyta) with a sensitivity analysis for bacterial contamination. Mar Biol 159: 2723-2729.

4. Li H, Durbin R (2009) Fast and accurate short read alignment with Burrows-Wheeler Transform. Bioinformatics 25:1754-60.

5. Gnerre S, Maccallum I, Przybylski D, Ribeiro FJ, Burton JN, et al. (2011) High-quality draft assemblies of mammalian genomes from massively parallel sequence data. Proc Natl Acad Sci U S A 108: 1513-1518.

6. Zerbino DR, Birney E (2008) Velvet: algorithms for de novo short read assembly using de Bruijn graphs. Genome Res 18: 821-829.

7. Finn RD, Bateman A, Clements J, Coggill P, Eberhardt RY, et al. (2014) Pfam: the protein families database. Nucleic Acids Res 42: D222-230.

8. Tatusov RL, Koonin EV, Lipman DJ (1997) A genomic perspective on protein families. Science 278: 631-637.

9. Benson G (1999) Tandem repeats finder: a program to analyze DNA sequences. Nucleic Acids Res 27: 573-580.

10. Grissa I, Vergnaud G, Pourcel C (2007) CRISPRFinder: a web tool to identify clustered regularly interspaced short palindromic repeats. Nucleic Acids Res 35: W52-57.

11. Lowe TM, Eddy SR (1997) tRNAscan-SE: a program for improved detection of transfer RNA genes in genomic sequence. Nucleic Acids Res 25: 955-964.

12. Piriyapongsa J, Rutledge MT, Patel S, Borodovsky M, Jordan IK (2007) Evaluating the protein coding potential of exonized transposable element sequences. Biol Direct 2: 31.

13. Stamatakis A, Ludwig T, Meier H (2005) RAxML-III: a fast program for maximum likelihood-based inference of large phylogenetic trees. Bioinformatics 21: 456-463.

14. Hofstetter V, Miadlikowska J, Kauff F, Lutzoni F (2007) Phylogenetic comparison of protein-coding versus ribosomal RNA-coding sequence data: a case study of the Lecanoromycetes (Ascomycota). Mol Phylogenet Evol 44: 412-426.

15. Wu M, Eisen JA (2008) A simple, fast, and accurate method of phylogenomic inference. Genome Biol 9: R151.

16. Williams TA, Foster PG, Nye TM, Cox CJ, Embley TM (2012) A congruent phylogenomic signal places eukaryotes within the Archaea. Proc Biol Sci 279: 4870-4879.

17. Katoh K, Misawa K, Kuma K, Miyata T (2002) MAFFT: a novel method for rapid multiple sequence alignment based on fast Fourier transform. Nucleic Acids Res 30: 3059-3066.

18. Capella-Gutierrez S, Silla-Martinez JM, Gabaldon T (2009) trimAl: a tool for automated alignment trimming in large-scale phylogenetic analyses. Bioinformatics 25: 1972-1973.

19. Darriba D, Taboada GL, Doallo R, Posada D (2011) ProtTest 3: fast selection of best-fit models of protein evolution. Bioinformatics 27: 1164-1165.

20. Miller MA, Pfeiffer W, Schwartz T. Creating the CIPRES Science Gateway for inference of large phylogenetic trees; 2010; New Orleans, LA, USA. pp. 1-8.

21. Fang G, Bhardwaj N, Robilotto R, Gerstein MB (2010) Getting started in gene orthology and functional analysis. PLoS Comput Biol 6: e1000703.

22. Poptsova MS, Gogarten JP (2007) BranchClust: a phylogenetic algorithm for selecting gene families. BMC Bioinformatics 8: 120.

23. Rosvall M, Bergstrom CT (2008) Maps of random walks on complex networks reveal community structure. Proc Natl Acad Sci U S A 105: 1118-1123.

24. Song N, Joseph JM, Davis GB, Durand D (2008) Sequence similarity network reveals common ancestry of multidomain proteins. PLoS Comput Biol 4: e1000063.

25. Marchler-Bauer A, Zheng C, Chitsaz F, Derbyshire MK, Geer LY, et al. (2013) CDD: conserved domains and protein three-dimensional structure. Nucleic Acids Res 41: D348-352.

26. Petersen TN, Brunak S, von Heijne G, Nielsen H (2011) SignalP 4.0: discriminating signal peptides from transmembrane regions. Nat Methods 8: 785-786.

27. Pluvinage B, Hehemann JH, Boraston AB (2013) Substrate recognition and hydrolysis by a family 50 exo-beta-agarase, Aga50D, from the marine bacterium Saccharophagus degradans. J Biol Chem 288: 28078-28088.
