# Supplementary material for: Genome Analysis of Planctomycetes Inhabiting Blades of the Red Alga Porphyra umbilicalis
Source: PLoS One. 2016 Mar 25;11(3):e0151883. doi: 10.1371/journal.pone.0151883 (PMC4807772; doi:10.1371/journal.pone.0151883)
Supplement: S2 Text — Additional results that supplement the findings presented in the main article are provided here. (DOCX) [file pone.0151883.s022.docx]

**Supplementary Results**

Genome Analysis of Planctomycetes Inhabiting Blades of the Red Alga *Porphyra umbilicalis*

JW Kim, SH Brawley, S Prochnik, M Chovatia, J Grimwood, J Jenkins, K LaButti, K Mavromatis, M Nolan, M Zane, J Schmutz, JW Stiller, AR Grossman

***1. Genome assembly validation:*** Scaffold sizes for P1 (8,470,422 bases), P2 (7,267,223 bases) and P3 (4,918,262 bases) fell within the range of known planctomycete genomes (3.8 Mb for *P. mikurensis* to 9.8 Mb for *G. obscuriglobus*) [[1-6](#_ENREF_1)] and contained an estimated 0.3-2.4% unidentified bases (Ns). To assess the completeness of the genomes, we confirmed the presence of 47 ‘universally conserved’ housekeeping genes [[7](#_ENREF_7),[8](#_ENREF_8)] in the P1, P2 and P3 assemblies. Furthermore, tRNA gene predictions for 29 bacterial genomes (**S3 Table**), including 22 species from the PVC superphylum, showed that P1, P2 and P3 each encode complete sets of tRNAs.

***2. Gene functions and gene-family content:*** In general, some members of the genus *Rhodopirellula* and P1 have relatively low densities of genes in gene families despite having relatively large genomes. Approximately 45 and 44% of the protein-coding genes in P1 and P2 occur in gene families, respectively; 2897 of 6382 for P1 and 2366 of 5409 for P2. At the other end of the spectrum, *K. stuttgartiensis* has a high percentage (56%) of genes in gene families despite its small genome. *Phycisphaera mikurensis, R. baltica* and P3 have the lowest gene family densities at 36%, 39% and 41%, respectively, with *P. mikurensis* having the smallest genome.

The largest gene families in the planctomycete genomes investigated are comprised of genes encoding response regulators (RR), serine/threonine protein kinases (STPK), sulfatases, sigma factors and a family of proteins containing the domain of unknown function 1559 (DUF1559), which are known to be unique to Planctomycetes. The response regulator family contains a diverse collection of two-component signal transduction elements that can be subdivided into proteins containing only receiver or phosphoacceptor domains (REC) such as CheY, OmpR, NtrC, PhoB and proteins that also contain a histidine kinase domain. P1, P2 and P3 have 90, 116 and 65 two-component signal transduction elements, respectively, that fall into the combined grouping (both just REC and also REC plus histidine kinase domain). These two-component regulators do not include all signal transduction systems; other response regulators such as those containing diguanylate cyclase (GGDEF) and forkhead association (FHA) domains, were placed into smaller gene-families by our classification algorithm. With respect to the STPKs, *G. obscuriglobus* has the greatest number at 110, while *P. mikurensis* and *K. stuttgartiensis* have only 4 and 2, respectively. P1, P2 and P3 have 111, 57 and 44 genes encoding various RNA polymerase sigma factors that show homology to sigma-70, sigma-54, and sigma-24 proteins. The number of genes in the ABC transporter family varied least among the highly represented gene families, and correlated well with genome size (Pearson *r* = 0.8, *p* = 0.0). On the other hand, the number of DUF1559 sequences was highly variable; *B. marina* has 208 while *K. stuttgartiensis* has 3. DUF1559 appeared to be specific to members of the PVC superphylum. P2 has the lowest number of DUF1559 genes among the *Rhodopirellula* at 45*.* DUF1549/1553 (related to planctomycete cytochrome c) and DUF1501 are also highly represented in most PVC organisms, although they were not found in *P. mikurensis* or *K. stuttgartiensis*. These two categories of DUF genes often occur in tandem with DUF1549/1553 directly 5’ of DUF1501; they are present 23 times as tandem sequences on the genome of P1. Sulfatases are also generally abundant in members of the genus *Rhodopirellula,* although they can also be abundant in other bacteria such as Lentisphaera araneosa, which has 302 sulfatases. On the other hand, the genome of the anammox organism *K. stuttgartiensis* encodes only 3 sulfatases.

Some gene families were not broadly represented across the analyzed planctomycete genomes, but were instead expanded in distinct genera or species. Members of the genus *Rhodopirellula* contain families of genes coding for adhesin-like transmembrane proteins. *R. rubra*, *R. maiorica*, and *R. sallentina* have the most copies (35, 31 and 34, respectively), while *R. baltica*, *R. europaea* and P2 have fewer (27, 24 and 21, respectively). The adhesin-like proteins associated with *Rhodopirellula* were not detected in large numbers in other Planctomycetes. The largest gene family in P3 has 137 members that mostly encode hypothetical proteins. Of these, many contain PEP-CTERM exosortase domains, which are short C-terminal sequences thought to be involved in protein export and sorting to various subcellular locations [[9](#_ENREF_9)]. P1 also has 6-8 families of genes containing PEP-CTERM domains, the largest having 25 members. The second largest gene-family in *B. marina* (150 members), annotated as hypothetical proteins, is found in low numbers in the *Rhodopirellula* (ranging from 2 to 3). In other Planctomycetes, this family has 13-68 members. There are 16, 2, and 13 members of this family in P1, P2 and P3, respectively.

We also surveyed the overall functional content of the P1, P2 and P3 genomes using high-level functional classification analysis provided in the Clusters of Orthologous Genes [[10](#_ENREF_10)] database [[11](#_ENREF_11)]. To investigate relationships between gene family expansion and function, we examined the distributions of COG domain hits across gene families of differing sizes. The proportional distribution of COG domain hits (representing gene function) in singletons (gene family size = 1) was fairly conserved across all 23 genomes examined (**Fig 2**), with the greatest number of singletons in the family ‘General Function Prediction Only’ (R), which is not surprising given the broad nature of that category. Many singletons are also associated with ‘energy production and conversion’ (C), ‘carbohydrate transport and metabolism’ (G), ‘translation, ribosome structure and biogenesis’ (J), ‘cell wall/membrane/envelope biogenesis’ (M) and ‘function unknown’ (S). A similar level of variation among the organisms was observed for COG domain hits in gene families with 2-5 members, again with the greatest number of family members being in ‘General Function Prediction Only’. More variation occurred for gene families containing 6 or more members, with the highest levels in the categories ‘signal transduction’ (T), ‘inorganic ion transport and metabolism’ (P) and ‘transcription’ (K). The largest range in distribution of COG domain hits across 23 genomes was in the category ‘inorganic ion transport and metabolism’ (P). The absolute distribution of COG domain hits for P1, P2 and P3 is shown in **S4 Fig (**with COG domain classes defined in **Fig 2b)**. While differences among the organisms are observed in a number of categories, perhaps the most striking differences are noted for the category ‘replication, recombination and repair’ (L) (e.g., compare *Z. galactanivorans*, *P. mikurensis*, *P. maris* and *P. limnophilus* with *L. araneosa*, *G. obscuriglobus* and *K. stuttgartiensis*).

***3. Sulfatase modifying factors***: Enzymes involved in sulfatase maturation, converting an active site cysteine to a formyl-glycine [[12](#_ENREF_12)], are also encoded on all three planctomycete genomes. P1, P2 and P3 have 7 (IMG: 2643312633, 2643313391, 2643315151, 2643315314, 2643315830, 2643313083, 2643316144), 7 (IMG: 2643291633, 2643293313, 2643292577, 2643294554, 2643290439, 2643294568, 2643291008), and 8 (IMG: 2603752414, 2603751911, 2603752256, 2603749573, 2603752317, 2603751988, 2603752621, 2603752670) genes encoding proteins with homology to sulfatase-modifying factors (SUMF) with FGE-sulfatase superfamily domains (PFAM), respectively. One P3 SUMF gene (IMG: 2603751911) is directly upstream of two full-length sulfatase genes (IMG: 2603751912, 2603751913) and is part of an operon-like gene cluster (**Fig 4b**) containing an α-L-fucosidase gene (IMG: 2603751914). Also in P2, a SUMF gene occurs in an operon-like gene cluster with an arylsulfatase gene. The other SUMF genes in P1, P2 and P3 do not occur within the general vicinity of sulfatase genes.

***4. Codon usage links degenerate tRNA gene clusters to genomic islands***: Organisms throughout all domains of life contain genomic regions densely populated by tRNA genes. Two types of such tRNA gene clusters are found in the planctomycete genomes that we investigated. One cluster contains 4 or 5 canonical tRNA genes (resembling functional tRNAs in primary and secondary structure) that are syntenic across all planctomycete genomes investigated. These canonical tRNA gene clusters mostly contain tRNA genes corresponding to highly used codons, including what is often the only tryptophan tRNA in a given genome. This cluster is conserved across the Planctomycetes and likely to be highly expressed based on codon usage and conservation of primary and secondary tRNA structure. Some member tRNA genes in this cluster may be co-transcribed with a translation elongation factor 1A (*ef1a*) gene that is embedded between canonical tryptophan and threonine tRNA genes (conserved gene order: Trp-*ef1a*-Thr-Gly-Tyr-Thr). On the other hand, degenerate tRNA gene clusters contain large numbers of partially degraded tRNAs, which are often acquired through HGT, and may be dispensable to the carrier organism [[13](#_ENREF_13),[14](#_ENREF_14)]. Such tRNA gene clusters in the planctomycete genomes vary substantially in size (2-28 tRNA genes) and appear to be evolving much more rapidly compared to their canonical counterparts, as evidenced by weak sequence similarity and disrupted synteny among species of the *Rhodopirellula*. P3 contains two large degenerate clusters (23 and 28 tRNA genes) which appear to have resulted from segmental duplication, but have since diverged. The largest clusters in P1 and P2 contain 14 and 10 tRNA genes, respectively. Most tRNA genes in degenerate clusters are degraded compared to other tRNA genes and thus have significantly lower Cove scores (*t*-test p-value < 0.0001 for P1, P2, and P3), which measure the degree to which the primary and secondary structures resemble a canonical tRNA gene. The exceptions are the isoleucine tRNA-UAU genes found in several Planctomycetes including P1, P2, P3 and all *Rhodopirellula*. These specific isoleucine tRNAs appear to be canonical in structure as indicated by high Cove scores (P1 - 66.39, P2 - 72.07, P3 - 77.42); a high Cove score suggests high tRNA activity. Notably, in the Planctomycetes, tRNA-UAU corresponds to the only codon that is represented exclusively within degenerate tRNA clusters (cannot be verified in *G. obscuriglobus* due to fragmented genome) and was thus likely introduced to the Planctomycetes through HGT. The conservation of primary and secondary structures as evidenced by Cove scores further suggests that tRNA-UAU is functionally active in several planctomycete genomes and likely confers more efficient translation of transcripts containing higher numbers of ‘AUA’ isoleucine codons.

Genomic islands (GI) are horizontally transmitted gene clusters that can facilitate adaptation to specific environments by conferring the carrier organisms with a selective advantage [[15](#_ENREF_15)]. GIs play central roles in pathogenesis as mobile reservoirs for virulence factors [[16](#_ENREF_16)] and in symbiosis as “expression islands” for genes needed for mutualistic exchange [[17](#_ENREF_17)]. Although most GIs eventually lose their mobility and become part of their host genomes, recently inserted GIs can be detected on the basis of abnormal GC content, dinucleotide composition, and codon bias, as well as the presence of mobile genetic elements [[18](#_ENREF_18)]. We analyzed P1, P2 and P3 for GIs using IslandViewer [[19](#_ENREF_19)], a genomic island detection tool that employs the strategies listed above. P1, P2 and P3 contained 1, 9 and 17 putative GIs that span 4.2, 187.1 and 248.7 kbp, respectively (positions of genomic islands are given in **S8 Fig**). P3 contained the most GIs despite having the smallest genome. This is not surprising given that P3 contains a large number of TE-associated genes (**Table 1**), which are often located in GIs. The majority of genes found within the GI regions of P1, P2 and P3 encode hypothetical proteins (179 of 373 total) and mobile elements including transposases and the inactivated derivatives XerD recombinases and integrases. Outside of these groups, which are the largest, GIs contained genes encoding a range of protein functions (**S3 Data**). Similar genes with associated functions sometimes occurred in the same GIs. One GI in P3 contained many genes encoding proteins likely involved in cell wall biogenesis; these included 7 glycosyl transferases, a sulfotransferase, a UDP-N-acetylmuramyl pentapeptide phosphotransferase (peptidoglycan synthesis), and 2 GDP-D-mannose dehydratases, which are involved in colanic acid synthesis. Another P3 GI contained 1 *pulE* and 3 *pulF* genes, which are involved in the secretion pathway for pullulanase, a type of glucanase that degrades the polysaccharide polymer pullulan. Notably, one of the 8 GH29 α-L-fucosidases found in P3 occurs on a GI. The GI gene clusters may play a role in adaptation. Codon usage analysis indicates that genes on GIs in P1, P2 and P3 have higher usage rates for ‘AUA’ isoleucine codons (corresponding to tRNA-UAU), which are rarely used overall (5.9, 2.4 and 3.1% of all isoleucine codons in P1, P2 and P3). This finding suggests that the isoleucine tRNA-UAU gene, likely acquired through HGT by the Planctomycetes, confers higher translation efficiency for horizontally acquired genes, which generally have higher occurrences of ‘ATA’. In essence, tRNA-UAU can facilitate expression of more recently acquired genes, increasing the rate at which new protein functions are obtained, which would allow more rapid adaptation to new environmental conditions.

***5. Selenocysteine-insertion and utilization genes in P1 and P2***: Selenocysteines (Sec) are co-translationally inserted into proteins by the selenosome complex [[20](#_ENREF_20)], which includes a Sec-specific elongation factor (SelB) and a dedicated tRNA (SelC) that recodes the opal (‘UGA’) stop codon for Sec insertion. Other proteins required for Sec-insertion include a Sec-specific tRNA synthase (SelA), and a selenophosphate synthetase (SelD), which catalyzes the synthesis of selenophosphate, the selenium donor for tRNA loading. P1 and P2 both contain full sets of genes required for Sec-insertion during protein synthesis (**S8 Table**). In addition, the P1 and P2 genomes contain genes encoding 2-selenouridine synthase (YbbB), which replaces the sulfur atom in 2-thiouridine with selenium at the wobble position of certain tRNAs (tRNA^Gln^, tRNA^Glu^_,_ tRNA^Lys^); the incorporation of selenium in these tRNAs enhance base pair discrimination, thus increasing translation efficiency [[21](#_ENREF_21)].

Genes involved in selenium utilization generally reside on two separate operons in Proteobacteria, the bacterial phylum that has the largest number of species with an identified selenoproteome. One operon consists of only Sec-insertion genes (*selA*-*selB*-*selC*) while the other includes genes involved in selenophosphate synthesis and utilization (*selD-ybbB*) (**Fig 6**) [[22](#_ENREF_22)]. The Sec-insertion genes in P2 display an unusual arrangement on the genome; the three protein-coding genes involved in Sec-insertion form an operon with a fourth gene of unknown function (*selB*-*unknown*-*selA*-*selD*), while the tRNA gene (*selC*) and *ybbB* occur at different positions on the genome (**Fig 6**). This operon arrangement is unique to P2 among all genomes in NCBI and is not co-localized on other Sec-utilizing Planctomycetes.

Sec-insertion genes are generally not well conserved among Planctomycetes and appear to have varied origins. For instance, individual genes of the P2 Sec-insertion operon encode proteins of distant sequence identity (33% to 46%) to homologs from various phyla; the closest known homologs for SelA are found in Firmicutes, for SelB in Proteobacteria, and for SelD in P1. The gene of unknown function within the sec operon encodes a protein with a transglut_core superfamily domain (PFAM01841), similar to that present on eukaryotic transglutaminases, which are believed to function as cysteine proteases in prokaryotes. The closest homolog for this putative cysteine protease occurs in P1, with more distant hits to proteins of Acidobacteria and Bacteroidetes; the gene encoding this protein is not present in other Planctomycetes. Furthermore, *selB* in P1, *G. obscuriglobus* and *I. pallida* appears to have been transmitted vertically, while P2 SelB shows weak sequence homology to proteins of other Planctomycetes (29% identity), with slightly more similarity to SelB in Proteobacteria (33% identity). Similarly, sequence comparisons suggest that the 2-selenouridine synthase genes (*ybbB*) in P1 and P2 may have been acquired independently from Bacteroidetes.

***6. Putative selenoproteins in P1 and P2***: Genes encoding putative selenoproteins were identified as described in the **Methods.** We found 7 and 2 selenoprotein genes in P1 and P2 that have a SECIS element, and 3 and 1 genes for which a SECIS element was not detected, but remain candidates based on homology to selenoproteins in other species. Putative selenoproteins identified in P1 and P2 are listed in **S9 Table**. In P1, a formate dehydrogenase alpha subunit (*fdhA*) is one of six putative selenoproteins with antioxidant activity. The proteins encoded by *fdhA* and *selD* may play a role in maintaining the Sec-decoding and insertion traits in bacteria [[23](#_ENREF_23)]. In Proteobacteria, *fdhA* is located in the vicinity of the Sec insertion operon (*selA*-*selB*-*selC*). In P1, *fdhA* forms an operon with *nuoEF*, genes that encode NADH:ubiquinone dehydrogenase I chains E and F (not Sec proteins). This P1 operon is well conserved (65% amino acid identity) in the myxobacterium *Plesiocystis pacifica* SIR-1 (isolated from beach seagrass, *Zostera* sp.) but not in any other genome (in NCBI). An *fdhA* phylogeny is shown in **Figure 7a**, which includes the 20 closest homologs (BLASTp against NCBI nr) to P1 *fdhA*. While *P. pacifica* is a Deltaproteobacteria, its *fdhA* is more highly conserved in amino acid sequence to P1 (71% identity) than to other Deltaproteobacteria *fdhAs*. Conversely, the genomes of other Planctomycetes such as the anammox bacterium *K. stuttgartiensis*, have an *fdhA* that more closely resemble those of other Deltaproteobacteria than those of *P. pacifica* and P1. Furthermore, *nuoEF* in P1 and *P. pacifica* shows greater sequence homology to the *nuoEF* in Actinobacteria than in Planctomycetes or Deltaproteobacteria.

**Figure 7b** shows a phylogeny of formylmethanofuran dehydrogenase subunit B (FmdB), which is encoded as a selenoprotein in P2, several Archaea, and two unclassified bacteria; glycine-containing homologs occur in three other Planctomycetes, *G. obscuriglobus*, *S. paludicola*, and *Z. formosa*. The closest match (71% amino acid identity) to the P2 FmdB belongs to an uncultured bacterium isolated as a fosmid from Lake Washington [[24](#_ENREF_24)].

**References for Supplementary Results**

1. Ankai A, Hosoyama A, Terui Y, Sekine M, Fukai R, et al. (2012) Complete genome sequence of *Phycisphaera mikurensis* NBRC 102666. EMBL/GenBank/DDBJ databases.

2. Klindworth A, Richter M, Richter-Heitmann T, Wegner CE, Frank CS, et al. (2014) Permanent draft genome of *Rhodopirellula rubra* SWK7. Mar Genomics 13: 11-12.

3. Richter M, Richter-Heitmann T, Klindworth A, Wegner CE, Frank CS, et al. (2014) Permanent draft genomes of the *Rhodopirellula maiorica* strain SM1. Mar Genomics 13: 19-20.

4. Richter M, Richter-Heitmann T, Klindworth A, Wegner CE, Frank CS, et al. (2014) Permanent draft genomes of the three *Rhodopirellula baltica* strains SH28, SWK14 and WH47. Mar Genomics 13: 13-14.

5. Wegner CE, Richter M, Richter-Heitmann T, Klindworth A, Frank CS, et al. (2014) Permanent draft genome of *Rhodopirellula sallentina* SM41. Mar Genomics 13: 17-18.

6. Jogler C, Waldmann J, Huang X, Jogler M, Glockner FO, et al. (2012) Identification of proteins likely to be involved in morphogenesis, cell division, and signal transduction in Planctomycetes by comparative genomics. J Bacteriol 194: 6419-6430.

7. Wu M, Eisen JA (2008) A simple, fast, and accurate method of phylogenomic inference. Genome Biol 9: R151.

8. Williams TA, Foster PG, Nye TM, Cox CJ, Embley TM (2012) A congruent phylogenomic signal places eukaryotes within the Archaea. Proc Biol Sci 279: 4870-4879.

9. Haft DH, Paulsen IT, Ward N, Selengut JD (2006) Exopolysaccharide-associated protein sorting in environmental organisms: the PEP-CTERM/EpsH system. Application of a novel phylogenetic profiling heuristic. BMC Biol 4: 29.

10. Finn RD, Bateman A, Clements J, Coggill P, Eberhardt RY, et al. (2014) Pfam: the protein families database. Nucleic Acids Res 42: D222-230.

11. Tatusov RL, Koonin EV, Lipman DJ (1997) A genomic perspective on protein families. Science 278: 631-637.

12. Dierks T, Schmidt B, von Figura K (1997) Conversion of cysteine to formylglycine: a protein modification in the endoplasmic reticulum. Proc Natl Acad Sci U S A 94: 11963-11968.

13. Puerto-Galan L, Vioque A (2012) Expression and processing of an unusual tRNA gene cluster in the cyanobacterium *Anabaena sp* PCC 7120. Fems Microbiol Lett 337: 10-17.

14. Levican G, Katz A, Valdes J, Quatrini R, Holmes DS, et al. (2009) A 300 kpb genome segment, including a complete set of tRNA genes, is dispensable for *Acidithiobacillus ferrooxidans*. Biohydrometallurgy: A Meeting Point between Microbial Ecology, Metal Recovery Processes and Environmental Remediation 71-73: 187-190.

15. Preston GM, Haubold B, Rainey PB (1998) Bacterial genomics and adaptation to life on plants: implications for the evolution of pathogenicity and symbiosis. Curr Opin Microbiol 1: 589-597.

16. Schmidt H, Hensel M (2004) Pathogenicity islands in bacterial pathogenesis. Clin Microbiol Rev 17: 14-56.

17. Uchiumi T, Ohwada T, Itakura M, Mitsui H, Nukui N, et al. (2004) Expression islands clustered on the symbiosis island of the *Mesorhizobium loti* genome. J Bacteriol 186: 2439-2448.

18. Juhas M, van der Meer JR, Gaillard M, Harding RM, Hood DW, et al. (2009) Genomic islands: tools of bacterial horizontal gene transfer and evolution. FEMS Microbiol Rev 33: 376-393.

19. Dhillon BK, Chiu TA, Laird MR, Langille MG, Brinkman FS (2013) IslandViewer update: Improved genomic island discovery and visualization. Nucleic Acids Res 41: W129-132.

20. Hatfield DL, Gladyshev VN (2002) How selenium has altered our understanding of the genetic code. Mol Cell Biol 22: 3565-3576.

21. Wolfe MD, Ahmed F, Lacourciere GM, Lauhon CT, Stadtman TC, et al. (2004) Functional diversity of the rhodanese homology domain: the *Escherichia coli* *ybbB* gene encodes a selenophosphate-dependent tRNA 2-selenouridine synthase. J Biol Chem 279: 1801-1809.

22. Zhang Y, Romero H, Salinas G, Gladyshev VN (2006) Dynamic evolution of selenocysteine utilization in bacteria: a balance between selenoprotein loss and evolution of selenocysteine from redox active cysteine residues. Genome Biol 7: R94.

23. Romero H, Zhang Y, Gladyshev VN, Salinas G (2005) Evolution of selenium utilization traits. Genome Biol 6: R66.

24. Vorholt JA, Kalyuzhnaya MG, Hagemeier CH, Lidstrom ME, Chistoserdova L (2005) MtdC, a novel class of methylene tetrahydromethanopterin dehydrogenases. J Bacteriol 187: 6069-6074.
